# The social production of the food environment: a mixed-methods analysis of the unequal distribution of capitals and competencies in a Latin American metropolis

**DOI:** 10.3389/fnut.2026.1823248

**Published:** 2026-07-10

**Authors:** Daniel Egana Rojas, Lorena Rodríguez-Osiac, Carolina Franch Maggiolo, Patricia Gálvez Espinoza

**Affiliations:** 1 Department of Primary Care and Family Health, College of Medicine, Transdisciplinary Group for Population Obesity (GTOP-UChile), Universidad de Chile, Santiago, San Miguel., Región Metropolitana, Chile; 2 Public Health School, College of Medicine, Transdisciplinary Group for Population Obesity (GTOP-UChile), Universidad de Chile, Santiago, Independencia, Región Metropolitana, Chile; 3 Department of Anthropology, College of Social Sciences, Transdisciplinary Group for Population Obesity (GTOP-UChile), Universidad de Chile, Santiago, Ñuñoa, Región Metropolitana, Chile; 4 Department of Nutrition, College of Medicine, Transdisciplinary Group for Population Obesity (GTOP-UChile), Universidad de Chile, Santiago, Independencia, Región Metropolitana, Chile

**Keywords:** food environments, food practices, habitus, Latin America, public health, social capital, social inequality, social practice

## Abstract

**Introduction:**

Food-environment models often privilege structural determinants and under-specify how eating practices and agency participate in (re)producing environments. This study analyzes, through a sequential explanatory mixed-methods design, how social eating practices are shaped by food environments and, in turn, contribute to their differential configuration across two districts of contrasting socioeconomic status. It proposes a theoretical framework that articulates Bourdieu's praxeology with Shove's theory of practices, conceptualizing food environments as social fields in which capitals are contested, and meanings, materials, and competencies are stabilized.

**Methods:**

Through a sequential explanatory mixed-methods design, the NEMS-P-Ch survey was administered to 785 adults, and eleven focus groups were conducted in two districts of contrasting socioeconomic status (SES) within a major Latin American metropolis.

**Results:**

In low-SES districts, provisioning practices center on farmers' markets and local shops, mobilizing social capital and budget-maximizing strategies to navigate material scarcity. Conversely, high-SES practices are dominated by supermarkets and digital platforms, guided by time efficiency and taste as markers of distinction.

**Discussion:**

The food environment is both a producer and a product of daily practices, which configure differentiated infrastructures based on the agents' trajectories and available capitals. Public policies must move beyond isolated individual and structural approaches, integrating the complexity of social practices to effectively address the persistent structural inequalities of urban food systems.

## 1 Introduction

During the 20th century, the notion of health underwent a major paradigm shift, moving from a deterministic conception, in which people's lives were subject to external forces, to one centered on personal choice and lifestyle (1). This change crystallized in the 1950s, when the idea of “lifestyle” began to be used to link personal habits to the risk of chronic diseases, emphasizing individual responsibility for one's own health (2). This perspective aligned with the evolution of public health food policies, which shifted from a message of “eating more” to prevent nutritional deficiencies, toward one of “eating less” to reduce the risk of chronic diseases such as heart disease and cancer (3). In this way, the “healthy lifestyle” was established as a pillar of neoliberal governance, in which rights are transferred to “rational” individuals who must make their own decisions, rendering invisible the structural conditions that constrain and shape those choices (4).

The first decades of the 21st century brought successive disruptions to the global food system. The 2008 financial crisis deepened inequalities in food access across Latin America. The COVID-19 pandemic (2020–2022) accelerated the digitalization of food purchasing and exposed the vulnerability of food supply chains to external shocks. More recently, the war in Ukraine (2022) drove sharp increases in global food and input prices. These events underscored the limits of purely individual approaches to food and nutrition, as their effects were unequally distributed across socioeconomic groups. By 2022, obesity prevalence among adults in Latin America and the Caribbean had doubled since 2000, reaching 29.9%—nearly twice the global estimate of 15.8% (5).

Faced with the limitations of the paradigm centered on individual responsibility and the lack of positive results in the population's food and nutritional health, a new paradigm emerged in public health (6, 7), shifting from merely individual responsibility to a more structural, population-based perspective. This shift in focus was consolidated around the notion of “obesogenic environments,” defined as environmental influences that promote obesity in the population, displacing the causes toward external factors that condition behavior and choices (8). Based on this idea, diverse conceptual frameworks on “food environments” were developed, seeking to account for how structural conditions—such as availability, access, cost, convenience regarding time and capabilities, and food quality—act as an interface mediating people's consumption decisions within the food system (6, 9–11).

The starting point was the concept of “obesogenic environment” by Swinburn et al. (8), which considers physical, economic, political, and sociocultural components at micro and macro scales, laying the foundations for understanding the environment as a force promoting obesity. Later models, such as the ecological frameworks of Glanz et al. (6) and Story et al. (7), organized these influences into domains (community, organizational, consumer) or nested layers that situate the individual at the center, but surrounded by physical and social environments that condition their possibilities. This deterministic perspective is reinforced by the assertion that obesity results from “people responding normally to the obesogenic environments that they find themselves in” (12).

Even when models analyze apparently personal factors such as food “desirability,” these are usually defined as functions of external influences, such as advertising and cultural norms. In fact, the model by Downs et al. (9) reinforces this view by explicitly separating individual preferences and tastes from the food environment, treating them as factors that only “interact” with an environment that pre-exists and conditions them.

Subsequently, more complex frameworks emerged, which, while introducing nuances, do not fundamentally alter this deterministic logic. The model proposed by Turner et al. (11, 13) represents a significant advance by distinguishing between an “external food environment” (the objective conditions of availability, prices, and marketing) and a “personal food environment” (the accessibility, affordability, convenience, and desirability experienced by the individual). The inclusion of a “personal” domain encompassing knowledge and preferences seems to grant greater weight to agency. Similarly, a food environment model developed in the Latin American context by Gálvez et al. (10) identifies five distinct environments (home, street, restaurant, supply, and organizational) where the daily routines of individuals and collectives unfold. Although research on food environments in Latin America has grown substantially in recent years, most studies remain concentrated on the structural dimensions of availability, access, and marketing (14, 15). However, in both cases, the focus of analysis remains on how the structural conditions of the external environment and the food system shape individual possibilities. What remains systematically invisibilized in the main food environment models are eating practices as such, that is, the social and cultural dynamics of cooking, commensality, and the construction of meanings around food (16). Also absent is the digital environment, whose role in shaping food practices has been increasingly recognized (17, 18).

The critique of food environment models regarding their lack of attention to sociocultural practices and dynamics is central to the work of Dalia Mattioni et al. (19, 20). Mattioni et al. (19) argues that the dominant approach, largely based on quantitative geospatial tools, offers a “limited integration of social aspects linked to people's daily paths and lifestyles.” This approach tends to treat individuals as if they respond passively to the availability and density of sales points, without satisfactorily explaining why they choose to buy in certain places rather than others. By doing so, Mattioni et al. (19), following Cummins (21), posit that the fundamental question of “how ‘environment' gets into the ‘body”' and translates into habits remains unanswered (19).

To overcome this limitation, Mattioni et al.'s (19, 20) conceptual proposal is to adopt practice theory as an analytical framework. The practice theory used by Mattioni et al. follows the development outlined by Shove et al. (22). These authors participate in the “practice turn” posited by Schatzki (23, 24), which indicates that the individual is socially constituted by the practices in which they participate. In Shove et al.'s (22) version, the focus is shifted from individual “behavior”—often understood as a series of rational choices—toward social “practice,” conceived as a routine and collective action that integrates different interdependent elements: materials (objects, technologies, and physical infrastructure), competencies (skills and practical knowledge), and meanings (ideas, aspirations, and symbolic values).

While the model by Shove et al. (22) allows focusing on the dynamics of practices and their reconfiguration, its application as an exclusive framework for analyzing food environments presents significant limitations. By displacing the individual and their embodied dispositions [Bourdieu's habitus (25)] as the generating principle of practices, the model risks underestimating the historical depth and inertia of social dispositions. For Bourdieu (25, 26), practice is not just a contingent configuration of elements, but the manifestation of a habitus—of a collective history made body—that generates a “practical sense” and a deeply rooted “taste.” Thus, resistance to adopting certain practices (even when materials and competencies are available) may reflect prior social dispositions rather than a lack of adequate meanings. Shove, by focusing on the dynamics of change, does not easily allow for accounting for the mechanisms of resistance and systematic reproduction of food inequalities. It is important to distinguish Bourdieu's use of “lifestyle” from the public health notion criticized above. In public health discourse, lifestyle implies individual choice and responsibility, obscuring structural conditions. In Bourdieu's framework, by contrast, lifestyles are systematic products of habitus—dispositions that generate apparently individual “choices” adjusted to material conditions (27).

Thus, for the analysis of eating practices, this work attempts to articulate Bourdieu's concepts of habitus, field, and capital (25, 26, 28) with the practice theory developed by Shove (22, 29). We understand food environments as fields: structured social spaces of forces and struggles. In these fields, agents with different volumes and types of capital (economic, social, cultural, symbolic) dispute the legitimacy of definitions of eating practices. This struggle unfolds over how to establish and legitimize the connections between the elements that, following Shove, constitute a social practice (materialities, competencies, and meanings). The connection between the individual and these socially structured practices is twofold. On the one hand, individuals are recruited by practices to carry them out (performance) actively and repeatedly. On the other hand, individuals internalize practices through habitus, understood as a system of durable and transferable dispositions incorporated throughout the agent's trajectory within the field. In articulating both proposals, habitus could explain why one social group feels an affinity for and adopts certain eating practices while another does not.

In this sense, as we have indicated in previous works, food environments are not configured homogeneously across heterogeneous social groups, reflecting patterns of urban segregation, territorial exclusion, and historical structural inequalities (30). The proposal of this work is to link this diversity of configuration to differential practices. The objective of this article is to analyze, through a sequential explanatory mixed-methods design, how social eating practices are shaped by food environments and, in turn, contribute to their differential configuration across two districts of contrasting socioeconomic status (SES) in a Latin American metropolis. Specifically, the study compares the distribution of capitals, competencies, and practices between a high-SES and a low-SES district to understand how structure and agency jointly produce differentiated food environments. This design was adopted because quantitative survey data can characterize the structural configuration of food environments, whereas qualitative inquiry is required to access the social practices and meanings that agents deploy within them.

In this way, we seek to deepen understanding of the social determination of food by addressing the following question: In what ways are eating practices a product of food environments, and how do they configure them differently across two districts with different SES? Guided by this theoretical framework, the study is oriented by the following analytical proposition: social eating practices are not solely a product of the structure of food environments but, in their routine exercise, actively contribute to their differential production and configuration across socioeconomic strata.

## 2 Materials and methods

### 2.1 Methodological design

The research employed a sequential explanatory mixed-methods design (31). The quantitative phase followed a non-experimental, cross-sectional approach and was oriented toward characterizing the structural and objectified dimensions of food environments, while the qualitative phase employed focus groups to explore social practices and subjective dispositions. The mixed methods approach was adopted with a purpose of complementarity, seeking to integrate (32) two levels of analysis to obtain a more comprehensive understanding of the relationship between structure and practice. Fieldwork was conducted in two districts of Santiago (Chile), selected for their distinct level of multidimensional poverty. Ñuñoa represents the high socioeconomic status (SES) district and Conchalí the low-SES district (Supplementary Figure S1). Both districts exhibit high internal socioeconomic homogeneity: 99.2% of participants in Ñuñoa belong to the upper-middle class, while 100% of participants in Conchalí belong to the middle and lower-income classes (30). Methodological details can be found in Gálvez et al. (33).

The study protocol was approved by the Ethics Committee of the Faculty of Medicine of the University of Chile (#013-2023, May 26, 2023), and all participants provided informed consent before data collection.

### 2.2 Quantitative phase: perceived Nutrition Environment Measures Survey (NEMS-P-Ch)

Quantitative data were collected using an abbreviated version of the Nutrition Environment Measure Scale-Perceptions questionnaire adapted for Chile (NEMS-P-Ch), which had previously been validated in the local population (34). The instrument, composed of 31 questions, was designed to evaluate individuals' perceptions of their food environments and the practices they develop within them, starting with the home environment. The version used was structured into six sections: 1) home food environment; 2) supply food environment; 3) restaurant food environment (including eating out and street food); 4) institutional/organizational food environment (workplace or place of study); 5) self-perception of body weight, health, and diet; and 6) general household background.

The study participants were adults aged 18 or older residing in the urban area of a Latin American metropolis who made some or all of their household's food purchases. The survey was administered to a random sample of two districts, selected based on government statistical data on multidimensional poverty (35). The sample size calculation assumed an infinite population for each district, unknown proportions, and a 5% error, with a normal distribution, resulting in a target sample of 384 people per district, for a total of 768 participants. Data collection was carried out by the Microdata Center of the University of Chile, which administered the instrument in person between October and December 2023. Finally, the questionnaire was successfully administered to 785 people, of whom 388 (49.4%) resided in the low socioeconomic status district.

A detailed description of the quantitative sample has been published elsewhere (30). Briefly, the sample comprised 785 participants (388 in the low-SES district, 397 in the high-SES district), with a majority of women (58.6%). Median age was 55 years in the low-SES district and 47 in the high-SES district. Educational attainment and socioeconomic group differed significantly between districts: 88.4% of participants in the high-SES district had more than 12 years of education compared to 36.3% in the low-SES district, and 99.2% of high-SES participants belonged to the upper-middle class, while 100% of low-SES participants belonged to the middle and lower-income classes (30).

Quantitative data analysis was conducted using descriptive statistics to present the frequency distributions of variables across districts. To compare the distinct socioeconomic strata, the Pearson Chi-square test (x^2^) was employed. When the assumptions of this test were not met, the Monte Carlo simulation method was used. When significant global differences were found, a *post-hoc* analysis of adjusted standardized residuals was performed. A significance level of *p* < 0.05 was established for all tests. It is important to specify that the significant statistical patterns and differences identified in this phase were subsequently subjected to an interpretation process in light of the proposed theoretical framework, using them as indicators of the objectified dimensions of the field (objectified capital) and of the dispositions incorporated by the agents (habitus), without this implying an *a priori* operationalization of said constructs in the instrument. The interpretive framework linking theoretical constructs to the empirical evidence is summarized in Supplementary Table S1.

### 2.3 Qualitative phase: focus groups

The intermediate stage of the research was developed through the realization of eleven (11) focus groups. The purpose was to analyze the meanings participants attribute to their functioning within food environments. Participant selection was anchored in the main criterion of the study: belonging to one of the two districts selected for their contrasting socioeconomic levels (high SES and low SES). Based on this fundamental division, group composition was defined to achieve internal homogeneity, combining three social determinants: employment status (people with and without paid work), nationality (Chileans and migrants), and gender. This strategy enabled the configuration of specific discussion profiles. Participant ages ranged from 19 to 84 years, with a mean of 55.4 years in Conchalí and 48.2 years in Ñuñoa (Tables 1, 2). Occupational profiles reflected the socioeconomic composition of each district: Ñuñoa participants included a higher proportion of professionals and individuals with tertiary education, whereas participants in Conchalí were predominantly homemakers, retirees, and informal workers, with more fragmented employment trajectories.

**Table 1:** Focus group profiles.

Focus group No.	Group profile	SES (District)	No. of participants
1	Women	Low SES	8
2	Women	High SES	5
3	Non-workers^*^	Low SES	7
4	Non-workers^*^	High SES	8
5	Men	Low SES	5
6	Men	High SES	4
7	Migrants	High SES	11
8	Migrants	Low SES	8
9	Workers^*^	High SES	8
10	Men	High SES	8
11	Workers^*^	Low SES	7

The focus group of men in the high SES district was repeated (focus group 4 and 10), due to the low turnout of the first one. ^*^Workers = participants with paid employment.

**Table 2:** Sociodemographic characteristics of focus group participants, by district.

Variable	Conchalí (Low-SES)	Ñuñoa (High-SES)
*N*	34	44
Female, % (*n*)	67.6 (23)	61.4 (27)
Age, mean	55.4	48.2^a^
Age, median	60	48
Chilean, % (*n*)	76.5 (26)	70.5 (31)

^a^ Age data unavailable for 1 participant in Ñuñoa (mean calculated from *n* = 43).

Focus group participants were purposively selected from the quantitative survey sample (36); when necessary, community members were also contacted. To mitigate social desirability bias, focus groups were conducted after the survey period by facilitators distinct from the survey team (37). The groups were conducted in community venues in each district and facilitated by four research assistants hired by the project (three anthropologists and one psychologist). Sessions lasted up to 90 min and included between four and eleven participants. Participants received 10,000 Chilean pesos (approximately 10 USD) as compensation. Each session was audio-recorded and fully transcribed for subsequent analysis.

Qualitative data analysis was conducted by the authors with the support of a research assistant, using a deductive thematic analysis (38) guided by the study's theoretical-conceptual framework. The aim was to generate categories from the data, using the theoretical constructs of Bourdieu (habitus, field, capital) and Shove et al. (materials, competencies, meanings) as a predefined coding system. The analytical process consisted of systematically reading transcripts to identify concrete manifestations of these theoretical categories in the participants' discourses. In this way, the analysis focused on applying the conceptual framework to organize, interpret, and deepen the meanings actors attribute to their provisioning and consumption practices within their respective food environments, comparing results from the high SES district with the low SES district.

To ensure analytical rigor, a researchers' triangulation procedure was implemented in two stages. In the first stage, four trained research assistants (three anthropologists and one nutrition scientist) independently coded all focus group transcripts in Atlas.ti v25 (39). The project director (nutrition sciences) coded a subset of transcripts to verify code functionality, then compared individual coding outputs and facilitated consensus discussions, resulting in a consolidated set of codes. In the second stage, the full research team (four authors: two anthropologists, one nutritionist, and one public health physician) reviewed the consolidated codes, defined the analytical categories, and agreed on the final thematic structure. This two-stage triangulation process strengthened the credibility and dependability of the qualitative analysis.

Finally, the integration of both phases employed methodological triangulation, oriented toward explanatory expansion rather than convergent validation (40). This analytical procedure implied a systematic articulation between the objectified indicators of the food environment—understood as the structure of material possibilities—and the practices, meanings, and dispositions emerging from participant discourses. This strategy investigated the zones of complementarity and tension between the methodological perspectives, enabling the construction of inferences about the recursive relationship between structure and agency. The correspondence between the main quantitative patterns and qualitative findings is synthesized in a Joint Display (Table 3).

**Table 3:** Joint display of quantitative and qualitative findings, by thematic dimension.

Dimension	Quantitative evidence — NEMS-P	Qualitative evidence — Focus groups	Structure↔practice relationship
**Supply environment**	Supermarket use: high-SES 93.7% vs. low-SES 85.3%; farmers' market: low-SES 85.3% vs. high-SES 55.2% (p < 0.005). Most important place: supermarket 73.6% high-SES vs. 51% low-SES; farmers' market 31.4% low-SES vs. 18.1% high-SES (p < 0.005). (Table 5; Supplementary Table S4)	“I go to the market for that, because I like it, I find it pleasant… it's like a social life, going to the market is spectacular, it's a good walk” (Man, Focus Group 5). “For me shopping is a chore, okay. I live super close to the Unimarc… I have the supermarket mapped, I buy what I have to buy, I've done it and I leave” (Woman, Focus Group 9)	Differentiated commercial infrastructure both shapes and is shaped by distinct provisioning practices: socialization and social capital in low-SES; efficiency and anonymity in high-SES
**Front-of-package labeling**	Does not buy foods with seals: low-SES 13.7% vs. high-SES 5.8% (p < 0.005). “Should not buy”: low-SES 32.2% vs. high-SES 19.6% (p < 0.005). (Table 4; Supplementary Table S3)	“They can tell me: ‘ma'am, but it is your health.' Yes, it is my health, but what I have in money, I have to invest in the children” (Woman, Focus Group 1)	The sanitary norm is incorporated in both groups, but its practical application is mediated by available economic capital: radical prohibition in low-SES that collides with material reality; flexible navigation in high-SES
**Commensality**	Family lunch “always”: low-SES 30.4% vs. high-SES 17.6% (p < 0.005). Once “always”: low-SES 46.1% vs. high-SES 23.4% (p < 0.005). (Supplementary Table S9)	“When we sit down, there is no phone, there is nothing… the table is to share and dialogue” (Woman, Focus Group 1). “Breakfast is like more or less separately… at night, we get together and have once” (Man, Focus Group 6)	Eating together follows distinct logics: daily moral value and family cohesion in low-SES; individualized meals with planned shared gatherings in high-SES
**Domestic food practices**	Non-traditional fruits available: low-SES 28.4% vs. high-SES 44.8% (p < 0.005); non-traditional vegetables: low-SES 27.3% vs. high-SES 43.1% (p < 0.005). Cooked vegetable consumption 3–6 × /week: high-SES 28.5% vs. low-SES 17.3% (p < 0.005). (Table 7; Supplementary Table S7)	“If there are no lucas to eat a steak, one has to eat what there is” (Woman, Focus Group 1). “I am not crazy about meat… I make everything with soy protein… I don't mess with your diet, I tell her, you don't mess with mine” (Woman, Focus Group 2)	Culinary practices contrast sharply: adaptation to scarce and fluctuating resources in low-SES; management of personal preferences and health projects in high-SES, enabled by diversified provisioning
**Physical access and perceived environment**	Reaches main purchase place in ≤ 10 min: high-SES 60.5% vs. low-SES 47.9% (p < 0.005). “Always” easy access to fresh produce: high-SES 76.1% vs. low-SES 59.8% (p < 0.005); “Very difficult”: low-SES 7.2% vs. high-SES 0.8% (p < 0.005). (Table 6; Supplementary Tables S5, S6)	“I live super close to the Unimarc… in 10 minutes I have the supermarket mapped, I buy what I have to buy, I've done it and I leave” (Woman, Focus Group 9). “I don't go to the supermarket, because it is very expensive. You go to the alley over there and noodles are two packages for a thousand pesos” (Woman, Focus Group 1)	Physical access is perceived as more favorable in high-SES, where proximity combines with planning competencies. In low-SES, despite longer travel times, daily purchasing at neighborhood stores compensates for distance to main supply points

### 2.4 Researcher positionality

Three of the four principal investigators reside in the high-SES district, which, together with the team's institutional position as university-based researchers, established a structural distance from low-SES communities. During fieldwork, this distance was mitigated through collaboration with local community leaders in both districts. The four authors (trained in anthropology, nutrition, and public health) brought different emphases to the interpretation of participants' discourses: while social science researchers attended primarily to the dynamics of practices, public health and nutrition researchers focused on structural conditions and policy dimensions. All team members have prior research experience in food environment studies. Interpretative differences were resolved iteratively through systematic comparison of independent coding outputs and consensus discussions.

## 3 Results

Based on the results of the NEMS-PCh and the focus groups, it is possible to initiate the analysis and reconstruct some dimensions of practice that have been objectified (in material conditions and objects) and incorporated (in dispositions, competencies, meanings, and perception schemes) in the social field of the food environment. Within what we might call incorporated dispositions, the attitude toward food purchasing stands out. When deciding what to buy in the supply food environment, people from the low SES district have greater sensitivity to price (79.1% vs. 57.7%, *p* < 0.005) and ease of preparation (61.9% vs. 39.5%, *p* < 0.005) than people from the high SES district, and also to weight control (67.5% vs. 40.1%, *p* < 0.005) (Supplementary Table S2). While price and ease of preparation (which can also be understood as time scarcity) account for material constraints, the issue of weight control may reflect the incorporation of the nutritional discourse of primary health care, which translates into nutritional self-surveillance. In a context with a higher prevalence of chronic diseases, “weight control” becomes a sanitary guideline that becomes explicit at the moment of purchase.

This is consistent with another element that accounts for the incorporation of nutritional self-surveillance, namely, the attitude toward the front-of-package warning labels (FOP) system, which, in the context of this study, are octagonal black seals with the message “High in” (calories, saturated fats, sugars, sodium). When asked whether their presence influences them, the low SES district population adopts more radical attitudes, indicating—almost three times more (13.7% vs. 5.8%, *p* < 0.005)—that they do not buy them. Facing this attitude, the high SES district population follows less drastic practices such as buying less of what they would have bought if it didn't have seals (29.5% vs. 14.2%, *p* < 0.005) (Table 4). That is, faced with a food with a seal, the low SES population perceives that they should not buy those foods (32.2% vs. 19.6%, *p* < 0.005). On the other hand, a higher proportion of high SES participants states that they should buy it fewer times (29.0% vs. 18.6%, *p* < 0.005) (Supplementary Table S3).

**Table 4:** Influence of front-of-package warning labels on food purchasing decisions (question 12), by district.

Response	High SES	Low SES	*p*-value
a. Yes, it influences; I choose foods with fewer warning labels	32.7% (130)	37.4% (145)	< 0.005^*^
b. Yes, it influences; I do not buy foods with warning labels	5.8% (23)^b^	13.7% (53)	
c. Yes, it influences; I buy less than I would have bought if the product had no warning labels	29.5% (117)^a^	14.2% (55)	
d. Not influence	32.0% (127)	34.8% (135)	

^*^Chi-Square. The subscript **a** indicates that the observed frequency is significantly higher than the expected frequency. The subscript **b** indicates that the observed frequency is significantly lower than the expected frequency (*p* < 0.05). Percentage (frequency).

Complementarily, the focus groups reveal how these attitudes are negotiated in daily life. The discourses account for a certain tension agents experience regarding sanitary norms and discourses on healthy eating. In the low SES, a stronger incorporation of the medical-sanitary discourse is observed. However, this knowledge (a form of cultural capital) collides directly with material reality. The competence to follow a prescribed diet is annulled by the lack of economic capital, generating a practice of resignation where the need of others, especially children, is prioritized:

“They can tell me: 'ma'am, but it is your health'. Yes, it is my health, but what I have in money, I have to invest in the children” (Woman, Focus Group 1).

The objectified dimension of the supply food environment, understood as the materiality structuring purchasing practices, reveals the existence of a markedly different field of possibilities between both districts. The configuration of objectified capital manifests, firstly, in a dissimilar commercial infrastructure: in the low SES district, purchasing practices are significantly more oriented toward the *feria libre* (farmers‘ market) (85.3% vs. 55.2%, *p* < 0.005), the wholesale market (16.2% vs. 3%, *p* < 0.005), neighborhood stores, corner shops, greengrocers (45.1%, vs. 37%, *p* 0.021), butcher shops (34% vs. 7.1%, *p* < 0.005), and bakeries (24.7% vs. 14.6%, *p* < 0.005). On the other hand, the supermarket, as the supply space, is significantly more present in the high SES (93.7% vs. 85.3%, *p* < 0.005) (Table 5). This difference is accentuated when asking about the most important place of purchase, where the supermarket is significantly higher in the high SES district (73.6% vs. 51%, *p* < 0.005), while in the low SES district the farmers' market (31.4% vs. 18.1%, *p* < 0.005) and the wholesale market (7% vs. 1.5%, *p* < 0.005) carry significantly greater weight (Supplementary Table S4). The configuration of commerce also translates into an experience of unequal access, as inhabitants of the high SES district indicate reaching their main supply place in 10 min or less with significantly greater frequency (60.5% vs. 47.9%, *p* < 0.005), in contrast to residents of the low SES district, who invest more time in their displacements (Supplementary Table S5).

**Table 5:** Food retail venues used for the majority of household food purchasing (question 7a), by district.

Location	High SES		Low SES		*p*-value
	Yes	No	Yes	No	
Supermarket	93.7% (372)^a^	6.3% (25)^b^	85.3% (331)	14.7% (57)	< 0.005^*^
Convenience store (OK Market, Gas station store)	1.0% (4)	99.0% (393)	1.0% (4)	99.0% (384)	
Bakery	14.6% (58)b	85.4% (339)^a^	24.7% (96)	75.3% (292)	< 0.005^*^
Corner store, grocery store, or greengroce	37.0% (147)^b^	63.0% (250)^a^	45.1% (175)	54.9% (213)	0.021^*^
farmers' market	55.2% (219)^b^	44.8% (178)^a^	85.3% (331)	14.7% (57)	< 0.005^*^
Wholesale market	3.0% (12)^b^	97.0% (385)^a^	16.2% (63)	83.8% (325)	< 0.005^*^
Butcher shop	7.1% (28)^b^	92.9% (369)^a^	34.0% (132)	66.0% (256)	< 0.005^*^
Other (please specify)	4.0% (16)	96.0% (381)	3.6% (14)	96.4% (374)	

^*^Chi-Square. The subscript **a** indicates that the observed frequency is significantly higher than the expected frequency. The subscript **b** indicates that the observed frequency is significantly lower than the expected frequency (*p* < .05). Percentage (frequency).

Said physical and access structure of the food environment shapes collective perceptions regarding the spectrum of possibilities offered by the district: for residents of the high SES district, the perception that it is ≪always≫ easy to access fresh fruit and vegetables (76.1% vs. 59.8%, *p* < 0.005) of good quality (69.8% vs. 56.2%, *p* < 0.005) and variety (67% vs. 58%, *p* < 0.005), as well as packaged foods without seals (26.7% vs. 8%, *p* < 0.005), is significantly higher (Table 6). These perceptions at the district level are confirmed when analyzing the experience inside the supply establishments, where residents of low SES districts perceive with greater frequency that it is ≪very difficult≫ (7.2% vs. 0.8%, *p* < 0.005) or ≪difficult≫ (9.3% vs. 3%, *p* < 0.005) to obtain fruits and vegetables, while in high SES districts the perception that it is ≪very easy≫ (62.7% vs. 45.6%, *p* < 0.005) is significantly superior (Supplementary Table S6).

**Table 6:** Residents' perceptions of food availability, quality, and variety in their neighborhood (question 6), by district.

Statement	High SES					Low SES					*p*-value
	Never	Occasionally	Almost Always	Always	DK/NA	Never	Occasionally	Almost Always	Always	DK/NA	
a. It is easy to buy fresh fruits and vegetables in my neighborhood.	1.2% (5)^b^	7.3% (29)^b^	15.4% (61)	76.1% (302)^a^	0.0% (0)	6.2% (24)	14.4% (56)	19.6% (76)	59.8% (232)	0.0% (0)	< 0.005^*^
b. The fresh foods sold in my neighborhood are of good quality.	0.8% (3)^b^	6.0% (24)^b^	22.9% (91)	69.8% (277)^a^	0.5% (2)	3.6% (14)	12.6% (49)	26.6% (103)	56.2% (218)	1.0% (4)	< 0.005^†^
c. A great variety of fresh fruits and vegetables can be found in my neighborhood.	2.0% (8)^b^	10.8% (43)	19.9% (79)	67.0% (266)^a^	0.3% (1)	8.5% (33)	14.4% (56)	18.8% (73)	58.0% (225)	0.3% (1)	< 0.005^†^
d. In my neighborhood, it is easy to buy packaged foods without any black warning labels (High in).	10.8% (43)^b^	26.4% (105)^a^	27.5% (109)^a^	26.7% (106)^a^	8.6% (34)	61.1% (237)	15.5% (60)	8.5% (33)	8.0% (31)	6.9% (27)	< 0.005^*^
e. The packaged foods in my neighborhood are of good quality.	1.8% (7)^b^	7.0% (28)^b^	26.9% (107)^b^	60.5% (240)^a^	3.8% (15)	9.3% (36)	18.3% (71)	36.9% (143)	30.4% (118)	5.1% (20)	< 0.005^*^
f. A great variety of packaged foods without any black warning labels (High in) can be found in my neighborhood.	14.1% (56)^b^	28.0% (111)^a^	26.4% (105)^a^	23.7% (94)^a^	7.8% (31)	61.9% (240)	17.3% (67)	6.4% (25)	7.7% (30)	6.7% (26)	< 0.005^*^

^*^Chi-Square.^†^Chi-Square (Monte Carlo) analysis of adjusted standardized residuals (Haberman's function). The subscript **a** indicates that the observed frequency is significantly higher than the expected frequency. The subscript **b** indicates that the observed frequency is significantly lower than the expected frequency (*p* < 0.05). Percentage (frequency).

While the survey data describe the material configuration of the supply environment, the discourses illuminate the practices and meanings that sustain it. From the point of view of discourses, practices of the supply environment in the low SES are organized around two complementary axes: on the one hand, the disposition (habitus) toward the management of limited economic capital; but on the other, the ability to articulate social capital as compensation for economic limitation, deploying specific competencies to function in the field. Thus, the farmers' market appears enunciated as the physical space (material) around which a significant part of provisioning practices is organized. The market emerges as a cultural space of socialization and interaction with different *caseros* (regular vendors):

“I go to the market for that, because I like it, I find it pleasant, I buy here, I buy there, I know the people, the *caseros*; this is the potato *casero*, this is the avocado one. So I go and visit them all, the fish ones, it's like a social life, going to the market is spectacular, it's a good walk” (Man, Focus Group 5).

However, and complementary to this dimension, the market is signified as the place where the budget can be maximized. The practice of going to the market, therefore, integrates a set of interdependent elements that constitute it as a practice. In addition to the material aspect of money and the products bought, competencies are put into play, such as knowing “how to make things stretch,” being able to evaluate the price-quality relationship, and articulating relationships cultivated with the different regular vendors. As one participant indicates:

“At least for me, they leave me five lucas (thousand pesos), and what do I do with five thousand pesos? My husband leaves me sometimes… he knows one buys a kilo of potatoes or a bit of carrot and so on. Sometimes, thank God, I've had *caseras* (female vendors), whom I tell just 500 pesos (5 USD) of carrots. Truly, I'm not ashamed to say it, but truly. Sometimes my husband doesn't have ten to leave me. Because there are people who go with ten, twenty thousand, and don't bring back, just a little. Fruit, sometimes I can't bring because imagine even fruit is extremely expensive.” (Woman, Focus Group 3).

Likewise, this practice is dominated, in this district, by certain transversal meanings such as necessity, saving, and subsistence. The purchasing decision transforms into a permanent negotiation between intention, ability, and possibility, which accounts for the practical sense of the actors:

“We go to whatever is cheapest. We go with an intention, but bring back something else. Because it depends, as the lady says, on the pocket, one wants... I'm going to go buy this, this other thing, and arrive at the market and find that it suits better to take this other thing, which will serve more than what you thought of doing” (Man, Focus Group 3).

A supply practice complementary to the weekly market is the daily purchase made at the neighborhood store. This practice is a central mechanism in daily subsistence and is strongly governed by immediacy and the management of fragmented, often insufficient economic capital. Unlike the weekly purchase at the farmers' market, which implies certain planning, even if basic, the purchase at the neighborhood store responds to day-to-day logic, to the urgency of resolving the immediate meal. As a participant relates, the practice is strongly conditioned by what economic capital permits, reducing itself to specific, unitary purchases or of few units:

“Normally an egg that one is going to buy, a tomato sauce that was missing. So suddenly something is missing or bread, which normally one buys in a neighborhood store. Three little breads, four little breads” (Man, Focus Group 3).

The neighborhood store constitutes itself as the privileged space for this practice, not only due to its physical proximity, but also because its business model adjusts to the logic of fractional consumption. Similar to the market, but with narrower limits, the principal competence agents enunciate is the ability to “stretch like gum” the daily budget. This implies buying the bare minimum, generally “something for the bread,” as a participant describes, or inputs for a single meal:

“They leave me seven thousand pesos daily, imagine. And I can't do more, because where is the poor man going to get it from. I stretch them like gum” (Woman, Focus Group 3).

In this sense, the practice is signified as the immediate resolution of need, leaving out any possibility of long-term planning, stockpiling, or speculation on offers. The daily purchase is a response to the question of what will be eaten “today,” contrasting with the logic of the supermarket, which is perceived as a space inducing spending and requiring capital that is not available. As a woman comments:

“I don't go to the supermarket, because it is very expensive. You go to the alley over there and noodles are two packages for a thousand pesos. In the supermarket a package of noodles is eight hundred pesos, nine hundred pesos” (Woman, Focus Group 1).

It is interesting to highlight that for actors of low SES, both the neighborhood store and the farmers' market constitute themselves as spaces of social relations. The figure of the “casero” (regular vendor) is relevant in both, since confidence and mutual knowledge can activate forms of social capital, such as *fiado* (purchasing on credit), which becomes crucial in the face of the tightness of daily economic capital. This network of local reciprocity is not present in spaces with greater institutionalization, like the supermarket:

“Here we already have *caseros* that even… some even have a pencil. We pay on the fortnight, sure (...) The baker, for example, passes by at four in the afternoon and, if you don't have money, you take the bread anyway. You pay afterwards” (Focus Group 3, man).

In contrast, in high SES districts, supply practices are governed by a different logic, where the greater amount of economic capital allows giving more space to cultural dispositions and negotiating the use of time. The supermarket, specialized stores, and home delivery purchases (mediated by digital platforms) appear as the primordial material infrastructure of the provisioning practice. In this group, the main competence is not stretching the budget, but efficient time management and the capacity to discern and access a certain “quality” in food. The supermarket offers the possibility of resolving multiple needs in a single place and moment, a practical logic of efficiency manifesting in the preference for proximity and ease of access (consistent with survey information), converting shopping into a “chore” that must be resolved with the greatest speed possible:

“For me shopping is a chore, okay. I live super close to the Unimarc [brand] on Chile-España [main street]. So I already know which are the aisles, what it has, what it doesn't have, so I go and in 10 minutes I already know, I have the supermarket mapped, I buy what I have to buy, I've done it and I leave” (Woman, Focus Group 9).

This practice centered on efficiency relies on planning competencies. The use of lists, whether mental or written, is a common tool to not “deviate much” from the plan and optimize the route. However, this control coexists with the temptation offered by the abundant supermarket supply. The practice of “walking all the aisles to remember what is missing” reveals a tension between planning and impulse buying, something only allowed by the existence of a looser budget:

“I make a list because sometimes I forget something; I still walk the whole supermarket, for example, to go remembering, to not make a list. But other times, when I go fast, there I do make a list to get out faster” (Woman, Focus Group 4).

Supermarket provisioning practices in the high SES also reflect the expression of cultural capital through taste. Product choice is not limited to basics, but extends to “niche” items, products for specific diets (vegan, gluten-free), imported foods (demanded by migrated communities), or specific brands functioning as markers of distinction. This capacity to discern and choose among a wide variety of products is a competence reflecting a specific habitus of the environment, and which often demands diversifying supply points to satisfy the need:

“I go to the Jumbo [supermarket chain] on Bilbao [main street], because it's not right next door, but it is the one that has the most vegan things. And from Jumbo I go to a greengrocer that is on the way and the other place where I buy a lot is a cafeteria in Plaza Sucre [neighborhood square] that is vegan and has bread, has tofu, has a bunch of things that serve me” (Woman, Focus Group 9).

Finally, the supermarket provisioning practice in the high SES extends to the digital realm. Online shopping, with home delivery or for pickup, adjusts to the previously referred disposition for efficient time management. This digital competence, practically absent in the low SES, allows externalizing the tedious work of selection and gathering, producing the idea that time is freed for other activities:

“We almost always buy at the super(market), we make like two or three online purchases and go to pick up, which is more comfortable than walking around” (Man, Focus Group 6).

The disposition to purchase centered on taste (cultural capital), and enabled by time and economic capital, allows that—in high SES districts—the farmers' market also appears as a supply complement, but resorting to a different practical sense. More than a necessity made possible by flexibility toward the budget, the market represents superior quality in fruits and vegetables:

“I find that supermarket vegetables are not good, and generally very green” (Woman, Focus Group 2).

The market would entail the desire for an “other flavor,” a manifestation of taste as incorporated cultural capital:

“My *cazuela* (stew) with celery from the market is different from the cazuela with celery that is frozen, or that was left several days in the freezer. No, it is an exquisite celery. The carrots are also crunchy carrots, those from the market” (Woman, Focus Group 2).

An idea that is complemented with a certain meaning of artificiality regarding supermarket fruits and vegetables:

“If one buys in the supermarket a fruit that lasts two, three months, it is a lot of additive, different than at the market” (Woman, Focus Group 2).

However, the farmers' market also appears as a complex space where practical sense is limited. Not all inhabitants of high SES districts possess the competencies (habitually present in the practices of low SES inhabitants) to buy at the market without being deceived with low-quality products. This would be a skill created in routine and reiterated practice, through the construction of social capital with the vendors. Its absence imposes a need for constant vigilance contrasting with the supposed transparency and standardization of the supermarket.

“When I had family, children and the whole thing, I went to the market, and the first times, if you don't make a *casero* (regular vendor), they give you pure rubbish, the first ones give you good and then you arrive home and find yourself with pure rubbish. So you have to have a *casero* to go to the market, if not you are fried” (Woman, Focus Group 4).

Differences in the objectified configuration of the supply environment in high and low SES districts, as well as the different competencies and meanings incorporated into practices, condition the material possibilities found in the home food environment. This becomes evident when analyzing differences in food availability in the home via the survey. Pantry composition constitutes a first level of differentiation: in low SES households, significantly lower access is verified to products functioning as markers of cultural and economic capital, such as ≪Fruits like mango, blueberries, blackberries≫ (28.4% vs. 44.8%, *P* < 0.005), ≪Vegetables such as arugula, watercress, Brussels sprouts≫ (27.3% vs. 43.1%, *P* < 0.005) and ≪Nuts without salt or sugar≫ (62.4% vs. 78.6%, *P* < 0.005). Conversely, in low SES households the presence of ≪Cured meats≫ (73.5% vs. 66.5%, *P* < 0.005) is significantly higher, while availability of ≪sweets and chocolates≫ (46.1% vs. 61.2%, *p* = 0.037), ≪ice creams≫ (33% vs. 42.3%, *p* = 0.007) and ≪salty snacks≫ (37.1% vs. 50.1%, *p* < 0.005) is lower (Table 7). This difference in access to inputs correlates with frequency of consumption, observing that high SES residents report consumption of ≪Vegetables, in stews or cooked≫ with a frequency of 3 to 6 times per week significantly higher than in low SES households (28.5% vs. 17.3%, *p* < 0.005) (Supplementary Table S7).

**Table 7:** Household food availability during the past week (question 1), by district.

Food item	High SES		Low SES		*p*-value
	Yes	No	Yes	No	
a. Fruits such as orange, banana, apple, pear, peach	97.0% (385)	3.0% (12)	94.8% (368)	5.2% (20)	
b. Frozen ready meals such as pizza, lasagna, empanadas	20.4% (81)	79.6% (316)	15.2% (59)	84.8% (329)	
c. Vegetables such as spinach, chard, broccoli, cauliflower, zucchini, beetroot	83.6% (332)	16.4% (65)	87.4% (339)	12.6% (49)	
d. Soft drinks (exclude unflavored mineral water)	59.9% (238)	40.1% (159)	62.1% (241)	37.9% (147)	
e. Fruits such as mango, blueberries, blackberries, raspberries, persimmon, pomegranate	44.8% (178)	55.2% (219)^a^	28.4% (110)	71.6% (278)	< 0.005^*^
f. Sweets (candies) and chocolates	61.2% (243)	38.8% (154)^a^	53.9% (209)	46.1% (179)	0.037^*^
g. Vegetables such as lettuce, cabbage, celery, tomato, onion, carrot	98.5% (391)	1.5% (6)	98.7% (383)	1.3% (5)	
h. Ice cream (tubs or individual)	42.3% (168)	57.7% (229)^a^	33.0% (128)	67.0% (260)	0.007^*^
i. Dairy such as milk (unflavored), fresh cheese, “quesillo,” yellow cheese, yogurt, or other unsweetened dairy.	94.7% (376)	5.3% (21)	94.3% (366)	5.7% (22)	
j. Unsalted or unsweetened nuts such as almonds, peanuts, and walnuts	78.6% (312)	21.4% (85)^a^	62.4% (242)	37.6% (146)	< 0.005^*^
k. Cold cuts (pork ham, salami, “jamonada,” pâté, etc.)	66.5% (264)	33.5% (133)^b^	73.5% (285)	26.5% (103)	0.033^*^
l. Legumes (beans, lentils, chickpeas, etc.) dried or canned	91.4% (363)	8.6% (34)	91.2% (354)	8.8% (34)	
m. Sweet or cream-filled cookies	49.6% (197)	50.4% (200)	53.1% (206)	46.9% (182)	
n. Processed meats or sausages (hot dogs, “longanizas,” “choricillos,” burgers, etc.)	57.7% (229)	42.3% (168)	53.4% (207)	46.6% (181)	
ñ. Vegetables such as arugula, watercress, Brussels sprouts, kale, endive, eggplant	43.1% (171)	56.9% (226)^a^	27.3% (106)	72.7% (282)	< 0.005^*^
o. Pastries, cakes, or sweet doughs	35.8% (142)	64.2% (255)	38.9% (151)	61.1% (237)	
p. Salty snacks (potato chips, corn sticks, or others)	50.1% (199)	49.9% (198)	37.1% (144)^a^	62.9% (244)	< 0.005^*^

^*^Chi-Square. The subscript **a** indicates that the observed frequency is significantly higher than the expected frequency. The subscript **b** indicates that the observed frequency is significantly lower than the expected frequency (*p* < 0.05). Percentage (frequency).

The qualitative data complement this household-level picture by revealing the practices and meanings through which these material conditions are experienced. In the low SES, foods acquired at the farmers' market and the neighborhood store—basic products, often with less variety and fluctuating quality—determine the repertoire of the available and of what is possible to cook, limiting the culinary repertoire and reinforcing dependence on simple and low-cost preparations. The pantry, in this context, more than a storage place is a rapid transit space. Culinary competence centers on the capacity to create a meal from very few ingredients, often bought minutes before, and therefore without long planning.

“I tell them there at the house, I tell them, there are times when there is and times when there isn't, and one has to get used to it like that. If there are no *lucas* (money) to eat a steak, one has to eat what there is.” (Woman, Focus Group 1).

The idea of “working wonders” with limited resources moves from the market stall to the home kitchen, dominating the meaning of the obligation to feed the family with “what there is.” This disposition, anchored in an imaginary of limited economic capital, is reactualized daily, generating continuity between past experience and the present:

“It's that one learned from childhood. We were 8 siblings where one bread had to be enough for everyone. A complete *marraqueta* (bread roll) was enough for the 8” (Man, Focus Group 3).

On the other hand, in the high SES, provisioning practices, centered on convenience and selective quality, structure a radically different domestic environment. The diversity of foods entering the home—from organic products to ingredients for specific diets—enable more complex and individualized culinary practices. The kitchen becomes a space for managing preferences and personal health projects, often fragmenting commensality:

“I am not crazy about meat, because also I got used to it with my daughter. I sometimes eat meat. But since she is vegan, then I make everything with soy protein. But yes, sometimes I get the urge to eat a little steak with rice. And she tells me, you are already cooking a broth. I don't mess with your diet, I tell her, you don't mess with mine.” (Woman, Focus Group 2).

In the high SES, eating practices of the domestic environment centered on self-care and health management stand out. Eating appears as a reflexive project, where cultural capital (knowledge about nutrition and specific diseases) and economic capital are mobilized to adapt practices to perceived bodily needs. The choice of foods and how to prepare them is a manifestation of this self-management, where pleasure and health meet in constant negotiation:

“I have irritable bowel and I've had that famous bacteria twice, Helicobacter pylori, which they call the killer bacteria. So, I am good for coffee, I smoke, so all those things are super irritating. So I am in that, trying to diet more, eat more vegetables, more things, healthier things.” (Woman, Focus Group 2).

The disparity in the pantry between high and low SES extends to the material and temporal conditions framing the act of eating. In low SES households, the evaluation of the ≪physical place where foods are consumed≫ as ≪very good≫ is significantly lower (27.1% vs. 49.1%, *p* < 0.005). The same occurs with the ≪time destined to eating≫, where the low SES perceives it as ≪very good≫ in a lower proportion (15.2% vs. 27.5%, *p* < 0.005), and the ≪schedules in which foods are consumed≫, where also the low SES perceives it to a lesser extent as ≪very good≫ (11.3% vs. 21.2%, *p* < 0.005) (Supplementary Table S8).

This configuration of the domestic environment articulates, in turn, with commensality practices revealing distinct social logics: although in low SES districts it is a significantly more frequent practice to ≪always≫ eat as a family during lunch (30.4% vs. 17.6%, *p* < 0.005) and *once* (tea-dinner) (46.1% vs. 23.4%, *p* < 0.005); this coexists with a greater propensity to not eat dinner (57% vs. 36.7%, *p* < 0.005) (Supplementary Table S9).

Additionally, this greater commensality occurs in a context where eating ≪always≫ in front of the television is a significantly more habitual practice during breakfast (31.4% vs. 18.6%, *p* < 0.005), lunch (33.2% vs. 19.7%, *p* < 0.005), and *once* (tea-dinner) (37.6% vs. 18.4%, *p* < 0.005) (Supplementary Table S10).

In parallel, the discourses illuminate the social meanings that accompany these commensality patterns. Family commensality in low SES districts emerges as a deeply rooted daily practice interpreted as a value, a meaning of cohesion persisting even when mediated by the materiality of television:

“In my house, when we sit down, there is no phone, there is nothing. With luck, when there are bad things, we watch the news if it's global, but beyond that, there isn't. Because the table, as we are at this moment, is to share and dialogue and say, you know what? This disagreed with me, I didn't like your food, whatever. But this (cellphone) kills communication and TV too.” (Woman, Focus Group 1).

Unlike in low SES, where family commensality has daily value, in high SES it is more fragmented and individual. Eating together is reserved for the weekend and usually constitutes a planned social event rather than an obligatory routine. The practice of eating alone reflects a more individualized and fragmented organization of time and domestic space: “Breakfast is like more or less separately, my dad has breakfast earlier, like at six a.m., my mom, suddenly like at ten, and me, well like at eight and generally it is like bread with butter, something fast like that, a coffee, or bread with egg and then to the U(niversity), and from there, at night, we get together and have *once* (tea-dinner)” (Man, Focus Group 6).

A joint display of the main quantitative and qualitative findings is presented in Table 4.

## 4 Discussion

The results of this study, developed in a context of high urban inequality in a Global South metropolis, support the analytical proposition outlined above: social eating practices are not solely the result of the structure of food environments, but, in their daily exercise, they actively contribute to configuring and differentiating them.

Our findings invite a review of the role agency plays in the construction and modification of food environments. For example, the *feria libre* (farmers‘ market) in the low SES is not only a possible supply option constituted as the preferred purchasing space, but people articulate a set of practices (know-how competencies of maximizing money, cultivating relationships with regular vendors, signifying it as a space of social life) that sustain it and give it meaning. The work of O'Meara et al. highlights the centrality of agency in the analysis of women controlling their food environment to improve their nutrition (41). Likewise, Neufeld et al., consistently with our results, have proposed in the study of adolescents that they shape their food environment in a reciprocal process, where their behaviors and choices form their environment at the same time they are shaped by it (42). The inclusion of agency in the analysis of food environments as a constructive, and not only reactive, gesture allows displacing the structural over-determination with which food environments have traditionally been understood (19, 20).

The farmers' market illustrates how structure and practice are co-constitutive. Purchasing there is not simply a matter of material access, economic meanings, and price-evaluation competencies. As the results show, the high SES population recognizes that it lacks the social competencies needed to navigate the market without being deceived. The figure of the “casero” (regular vendor) responds to a group habitus anchored in community interdependence. Its absence in the high SES population generates mistrust and constant vigilance so as not to be deceived. It is a sort of relational competence, given by mutual recognition. Munawaroh et al. (43) have studied this construction of social capital in traditional markets in Indonesia as a distinctive relational competence, product of the articulation of elements like familiar language, construction of sensory trust, personalized relationships, and shared cultural norms. In a similar line, Van Eck (44) analyzes how relational and affective practices are the central element in the persistence of a market in Amsterdam, over economic or access elements.

Regarding the question of how practices differentially configure food environments in two districts of different SES, the exposed results allow understanding that provisioning practices respond to differential logics. While in the low SES district said practices are dominated by economic pressure and the contingent maximization of scarce resources, in the high SES district provisioning practices appear to revolve around a conception of time optimization given by purchase efficiency and the diversification of specific foods as markers of social distinction. These results are consistent with proposals like those of Anigstein (2), who describes greater capacity for planning and routinization in Chilean women of higher SES, while food purchases of low SES women are based on daily actions, small amounts, and contingencies. The results are also consistent with international literature. As we have already seen, the results show that the supply environment is differentially organized based on the social relations each SES articulates. While in the low SES food purchasing practices are traversed by social relations, in the high SES the provisioning practice in the supermarket or on digital platforms orients toward anonymous and temporally efficient transactions. The need to have regular vendors to ensure quality, price, and, eventually, buy “on credit” (*fiado*) accounts for the centrality that social capital management fulfills in low SES people. These distinctions between modes of relation and exchange end up configuring two figures of consumers: on one hand, an actor inserted in a system of familiar and direct practices dependent on the generation of mutual bonds, and on the other, an independent client deploying a practical logic based on self-service in an anonymous environment (45).

A third element emerging in the comparative analysis of practices relates to the meaning time fulfills in the structuring of practices in food environments. While surveys, in the low SES, account for a time limitation, provisioning practices tend to be temporally more extensive, both due to distance to the habitual place of purchase, accounting for a different urban ordering, and due to the social and daily logic of provisioning. On the other hand, in the high SES, although there are fewer constraints, time appears as an element to be managed efficiently, “buying time” through strategies like the efficiency of the “mapped” supermarket and online purchases. The apparent paradox occurring in the low SES between time scarcity and provisioning practices consuming a large amount of time is consistent with literature, which has documented how the need to optimize a reduced budget forces parents to invest greater time in purchasing, by visiting multiple stores or traveling to distant but more affordable places (46). On the other hand, Vos et al. (47) have documented how in higher socioeconomic levels lack of time is conceptualized as a barrier that must be managed through strategies like planning purchases with a weekly supermarket list.

Another differentiating element between practices differentially organizing the food environment of low and high SES families has to do with commensality. Quantitative data show that, in low SES districts, the practice of eating together is significantly more frequent, which is reinforced in focus groups alluding to meanings accounting for an imaginary of cohesion and family obligation. This idea resembles that developed by Van Der Heijden and Wiggins (48), who see in the imperative to gather the family at the table a performative way of expressing socially validated identities, like the idea of “competent parents,” reaffirming the idea of a family duty even in a context of precarity. Conversely, in the high SES, greater fragmentation of work and study schedules is experienced, translating into more individualized commensality practices.

A last element emerging in the comparative analysis relates to the tensions produced by the sanitary discourse and its real possibilities of realization. Quantitative data indicate that in the low SES district there is greater incorporation of sanitary norms into daily dispositions. For example, they express a more radical attitude toward warning labeling, declaring in greater proportion that they avoid buying products with seals. However, qualitative data show the material limits this normative disposition encounters. Discourses of low SES participants show frustration between possessing the knowledge of what “one should eat,” but indicate limitations in economic capital to translate that competence into practice. In this sense, there is a conflict between incorporated meanings of the medical-sanitary discourse as a disposition to self-surveillance and the objective and material conditions of the food environment, which make the practice unviable. This does not occur in the high SES, where, although there is a more indulgent declaration, like the practice of “buying less” of products with seals, greater economic capital allows constructing a more nuanced, reflexive, and manageable self-care project. These data are consistent with international evidence accounting for structural barriers for the articulation of individual agency. McCoy et al. (49) report how health practices of SES populations are governed by affordability, imposing on them the adaptation of their habits to the only options that result economically viable. More broadly, this highlights the limitations of what has been termed agento-structural policies—interventions that seek to modify practice but require a significant amount of individual agency, conditioned by an unequal distribution of capitals, to achieve a positive effect on dietary health (50).

### 4.1 Limitations

Although the findings of this work align with other research, they must be understood in light of the limitations inherent to this study. The focus on two districts of a Latin American metropolis, while allowing understanding of the differential relationship between daily practices and the construction of food environments of distinct SES, does not allow extrapolating results and conclusions to the entire city or to regional contexts with other sociodemographic configurations, where food infrastructure and social practices operate under other logics. The transferability of findings to other urban contexts should be approached with caution, given the specific socio-territorial conditions of the studied metropolis, including its commercial infrastructure, migration patterns, and welfare arrangements. Furthermore, although these districts exhibit high internal socioeconomic homogeneity, intra-district variability cannot be ruled out entirely. Moreover, while the study design captured variation in the volume of capital across districts, it did not systematically differentiate intra-district positions defined by the structure of capital (for instance, agents rich in cultural capital but not in economic capital vs. those with the opposite composition), which Bourdieu's framework identifies as central to understanding variation in practices. Social desirability bias, inherent to research on food and health topics, may also have affected focus group responses despite the procedural measures adopted to mitigate it.

Additionally, the research team's institutional position as university-based researchers may have introduced power asymmetries during fieldwork, despite the use of community venues and non-author facilitators.

Likewise, as a cross-sectional design, this research has limitations for understanding the temporal evolution of these eating practices and dispositions, their social reproduction, and possible resistance to the transformations that food systems have experienced since the second half of the 20th century.

Linked to this, the focus on the relationship between practice and the food environment limits the analysis of the macrostructural determinants of the globalized food system. By privileging daily practices that articulate capitals, competencies, and meanings in a food field defined as an environment, this work fails to be sensitive to the social and commercial determinations that corporate and state actors deploy to model food availability and affordability in concrete forms such as supermarkets or farmers' markets. Future research should aim to incorporate this dimension into the analysis.

Despite these limitations, the study presents several noteworthy strengths. First, the sequential explanatory mixed-methods design enabled a systematic integration of quantitative and qualitative evidence under the purpose of complementarity. Second, the conceptual articulation between Bourdieu's praxeology and Shove's theory of practices offers a novel theoretical lens for food environment research, one that moves beyond both purely structural and purely individual approaches. Third, the quantitative phase employed random household sampling and reached a sample of 785 participants, providing robust statistical power for the between-district comparisons. Finally, by situating the study in Santiago, Chile, the research contributes empirical evidence from the Global South, a context that remains underrepresented in the food environment literature.

### 4.2 Public health implications

The results of this work have direct implications for the design and evaluation of public policies on food and nutrition, particularly in high-inequality settings. By conceptualizing food environments not as external structures but as fields co-produced by daily practices, this framework allows rethinking public policy by showing the limited efficacy of two predominant intervention approaches (individual and structural). On one hand, it helps understand the limited efficacy of public policies oriented toward purely structural interventions (for example, those operating by modifying availability or access to determined foods or points of sale), and which do not consider other elements of social practices (such as competencies and meanings); on the other hand, it accounts for the inefficacy of those interventions operating exclusively on the idea of agency, knowledge, or internalized competencies (for example, food and nutrition education), but which do not incorporate other elements (often structural) like material conditions and knowledges enabling said practices. The efficacy of an intervention, therefore, does not reside in the modification of a particular element, but in work on a set of factors that can be schematized as materials, meanings, and competencies, which would account for the incorporation of a practice.

Even well-designed structural policies can produce unequal effects when they rely on forms of agency that are unevenly distributed across socioeconomic groups. The front-of-pack warning label system in Chile illustrates this tension: low-SES participants adopted the sanitary norm more radically (“should not buy”), yet their capacity to enact it was constrained by economic capital, leading to frustration rather than behavioral change. High-SES participants, by contrast, navigated the same policy with greater flexibility, moderating consumption without renouncing entire food categories. This illustrates the limits of agento-structural policies—interventions designed to modify practices but whose effectiveness depends on a volume of individual agency that is itself conditioned by an unequal distribution of capitals (50).

In a district like the Low-SES, policies that strengthen the social infrastructure of farmers‘ markets—recognizing the role of regular vendors and informal credit—are more likely to succeed than interventions that assume supermarket access alone will improve diets. In the High-SES district, the findings reveal a gap between the sensory appreciation of fresh food quality and the relational competencies needed to navigate farmers' market environments without distrust. Policies that bridge this gap (for instance, by building transparency and trust into market spaces without requiring the long-term cultivation of personal vendor relationships) could activate the quality-driven dispositions that already exist. The contrast between the two districts illustrates a broader principle: effective food policy cannot be designed around a single model of consumption. It must be tailored to the specific practices, competencies, and social relations through which different groups procure, prepare, and consume food.

However, and against voluntarism, it is not always possible to modify these elements normatively. The history of social collectives has defined a tradition of reproduction of dispositions (habitus), which makes the adoption of practices, even when constitutive elements are present, not occur homogeneously. Even when structural policies are maintained (e.g., front-of-package warning labels), it is essential to stop thinking of universal agents (neutral and homogeneous), and identify how the food environment, as a field, operates on the practices the policy supposes and enables, transitioning toward more pertinent, territorial, and just policies.

## 5 Conclusions

Based on the objective posited, this work has shifted from a structuralist model, conceiving food environments as a set of external objective conditions, to an approach that empirically demonstrates that the structure of the field is both producer and product of daily social practices. In the low-SES district, social capital and budget-maximizing practices sustain a supply environment centered on farmers' markets and neighborhood stores; in the high-SES district, efficiency-oriented practices reinforce an environment dominated by supermarkets and digital platforms. The food environment is reflected dynamically and complexly in a set of practices that, interdependently, refer to materialities, competencies, and meanings.

This understanding depended on the complementarity of both methods. The survey data mapped the structural configurations of each food environment, while the focus groups revealed the competencies, meanings, and social relations through which agents navigate and reproduce those configurations. Taken together, they show that structure and practice are not sequential but co-constitutive.

To advance more effective public policies, approaches that recognize this interrelated dimension of the environment and eating practices are required. Understanding how people act within the limits of their environment and, at the same time, their practices produce them is key to designing fairer and more sensitive responses to the diverse and unequal realities of Latin American cities.

## Data Availability

The original contributions presented in the study are included in the article/Supplementary material, further inquiries can be directed to the corresponding author.

## References

[1] Pakholok O. The idea of healthy lifestyle and its transformation into health-oriented lifestyle in contemporary society. Sage Open. (2013) 3:2158244013500281. doi: 10.1177/2158244013500281

[2] Anigstein MS. Families' food provisioning strategies among households headed by working mothers in Santiago of Chile. Rev chil nutr (2019) 46:129–36. doi: 10.4067/s0717-75182019000200129

[3] Nestle M. Food Politics: How the Food Industry Influences Nutrition and Health. Rev. and expanded ed. Berkeley: University of California Press (2007). p. 486.

[4] Anigstein MS Ferrer-Lues M Watkins L Robledo MC Bosnich M. Being for others and material conditions: the limits of the “healthy lifestyles” notion for Chilean women. Medl Anthropol. (2021) 40:745–758. doi: 10.1080/01459740.2021.1935924 34229549

[5] FAO IFAD PAHO UNICEF WFP. Latin America and the Caribbean: A Regional Overview of Food Security and Nutrition. Santiago: FAO; IFAD; UNICEF; PAHO; WFP (2026).

[6] Glanz K Sallis JF Saelens BE Frank LD. Healthy nutrition environments: concepts and measures. Am J Health Promot. (2005) 19:330–3. doi: 10.4278/0890-1171-19.5.330 15895534

[7] Story M Kaphingst KM Robinson-O'Brien R Glanz K. Creating healthy food and eating environments: policy and environmental approaches. Annu Rev Public Health. (2008) 29:253–72. doi: 10.1146/annurev.publhealth.29.020907.090926 18031223

[8] Swinburn B Egger G Raza F. Dissecting obesogenic environments: the development and application of a framework for identifying and prioritizing environmental interventions for obesity. Prev Med. (1999) 29:563–70. doi: 10.1006/pmed.1999.0585 10600438

[9] Downs SM Ahmed S Fanzo J Herforth A. Food environment typology: advancing an expanded definition, framework, and methodological approach for improved characterization of wild, cultivated, and built food environments toward sustainable diets. Foods (2020) 9:532. doi: 10.3390/foods9040532 32331424 PMC7230632

[10] Gálvez P Egaña D Masferrer D Cerda R. Proposal for a conceptual model for the study of food environments in Chile. Rev Panam Salud Publica. (2017) 1–9. doi: 10.26633/RPSP.2017.169 31384280 PMC6650624

[11] Turner C Aggarwal A Walls H Herforth A Drewnowski A Coates J . Concepts and critical perspectives for food environment research: a global framework with implications for action in low- and middle-income countries. Glob Food Secur. (2018) 18:93–101. doi: 10.1016/j.gfs.2018.08.003

[12] Swinburn B Sacks G Hall KD McPherson K Finegood DT Moodie ML . The global obesity pandemic: shaped by global drivers and local environments. Lancet. (2011) 378:804–14. doi: 10.1016/S0140-6736(11)60813-1 21872749

[13] Turner C Kadiyala S Aggarwal A Coates J Drewnowski A Hawkes C . Concepts and Methods for Food Environment Research in Low and Middle Income Countries. London, UK: Innovative Methods and Metrics for Agriculture and Nutrition Actions (IMMANA) Programme (2017). Available online at: https://researchonline.lshtm.ac.uk/id/eprint/4656361/1/Turner%20et%20al%20FEWG_TechnicalBrief_low-4.pdf (Accessed January 15, 2026).

[14] Pérez-Ferrer C Auchincloss AH De Menezes MC Kroker-Lobos MF Cardoso LDO Barrientos-Gutierrez T. The food environment in Latin America: a systematic review with a focus on environments relevant to obesity and related chronic diseases. Public Health Nutr. (2019) 22:3447–64. doi: 10.1017/S1368980019002891 31666140 PMC10260576

[15] Mendes LL Rocha LL Botelho LV De Menezes MC Júnior PCPDC Da Camara AO . Scientific research on food environments in Brazil: a scoping review. Public Health Nutr. (2023) 26:2056–65. doi: 10.1017/S1368980023000836 37232243 PMC10564610

[16] Rivera-Navarro J Conde P Díez J Gutiérrez-Sastre M González-Salgado I Sandín M . Urban environment and dietary behaviours as perceived by residents living in socioeconomically diverse neighbourhoods: a qualitative study in a Mediterranean context. Appetite. (2021) 157:104983. doi: 10.1016/j.appet.2020.104983 33045303

[17] Granheim SI Løvhaug AL Terragni L Torheim LE Thurston M. Mapping the digital food environment: a systematic scoping review. Obes Rev. (2022) 23:e13356. doi: 10.1111/obr.13356 34519396

[18] Navarro JL Tudge JRH. Technologizing bronfenbrenner: neo-ecological theory. Curr Psychol. (2023) 42:19338–54. doi: 10.1007/s12144-022-02738-3 35095241 PMC8782219

[19] Mattioni D Galli F Brunori G. Understanding the food environment: the role of practice theory and policy implications. In:Burlingame B Dernini S, editors. Sustainable Diets: Linking Nutrition and Food Systems. UK: CABI (2019). p. 121–30. doi: 10.1079/9781786392848.0121

[20] Mattioni D Loconto AM Brunori G. Healthy diets and the retail food environment: a sociological approach. Health Place. (2020) 61:102244. doi: 10.1016/j.healthplace.2019.102244 31748171

[21] Cummins S. Neighbourhood food environment and diet—time for improved conceptual models? Prev Med. (2007) 44:196–7. doi: 10.1016/j.ypmed.2006.11.018 17222896

[22] Shove E Pantzar M Watson M. The Dynamics of Social Practice: Everyday Life and How it Changes. Los Angeles: SAGE (2012). p. 191.

[23] Schatzki TR Knorr-Cetina K Savigny E von (editors). The Practice Turn in Contemporary Theory. New York: Routledge (2001). p. 239.

[24] Schatzki TR. Social Practices: a Wittgensteinian Approach to Human Activity and the Social. New York: Cambridge University Press (1996). doi: 10.1017/CBO9780511527470

[25] Bourdieu P. Outline of a theory of practice. Buenos Aires: Prometeo Libros (2012). p. 1.

[26] Bourdieu P. The Practical sense. Primera edición de Siglo XXI en España. Madrid: Siglo XXI (2008).

[27] Bourdieu P. Distinction: The Criteria and Social Bases of Taste. Tercera edición: febrero de 2016, segunda reimpresión: noviembre de 2017. Madrid: Taurus (2017).

[28] Bourdieu P. General Sociology Course 1: Fundamental Concepts. Ciudad Autónoma de Buenos Aires: Siglo XXI Editores (2019). p. 1.

[29] Shove E (editor). The Design of Everyday Life. New York, NY: Berg (2007). p. 174.

[30] Franch Maggiolo C Egaña Rojas D Rodríguez Osiac L Villegas Ríos R Ortega Guzmán A Gálvez Espinoza P. Obesogenic urban food environments in Santiago, Chile: perceptions from a cross-sectional study of two socioeconomically distinct neighborhoods. Health Place. (2025) 94:103493. doi: 10.1016/j.healthplace.2025.103493 40479871

[31] Creswell JW Plano Clark VL. Designing and Conducting Mixed Methods Research. Third edition. Los Angeles, California, London, New Delhi, Singapore, Washington DC, Melbourne: Sage (2018). p. 492.

[32] Fetters MD Curry LA Creswell JW. Achieving integration in mixed methods designs—principles and practices. Health Serv Res. (2013) 48:2134–56. doi: 10.1111/1475-6773.12117 24279835 PMC4097839

[33] Gálvez P Rodríguez L Franch C Egaña D. Rethinking the social determination of food in Chile through practices and interactions of actors in food environments: nonexperimental, cross-sectional study. JMIR Res Protoc. (2024) 13:e62765. doi: 10.2196/62765 39270213 PMC11437218

[34] Molina P Villegas Ríos R Gálvez P Rodríguez L Egaña D. Adaptation and validation of the Perceived Nutrition Environment Measures Survey for the Chilean context (NEMS-P-Ch). Rev Chil Nutr. (2023) 50:371–81. doi: 10.4067/s0717-75182023000400371

[35] MIDESO. Metodologí*a de medición de pobreza multidimensional con entorno y redes*. Serie Documentos Metodológicos Casen N°32 (2016). Available online at: http://observatorio.ministeriodesarrollosocial.gob.cl/pobreza (Accessed January 15, 2026).

[36] Teddlie C Yu F. Mixed methods sampling: a typology with examples. J Mix Methods Res. (2007) 1:77–100. doi: 10.1177/1558689806292430

[37] Bergen N Labonté R. “Everything is perfect, and we have no problems”: detecting and limiting social desirability bias in qualitative research. Qual Health Res. (2020) 30:783–92. doi: 10.1177/1049732319889354 31830860

[38] Gibbs G. Analyzing Qualitative Data. 2nd edition. Los Angeles, London, New Delhi, Singapore, Washington DC, Melbourne: SAGE (2018). p. 1.

[39] ATLAS.ti Scientific Software Development GmbH. (2025). ATLAS.ti. Available online at: https://atlasti.com (Accesed March 3, 2026).

[40] Flick U. “Triangulation.” The SAGE Handbook of Qualitative Research. London: SAGE (2023). p. 444–61.

[41] O'Meara L De Bruyn J Hope T Fajó-Pascual M Hodge R Turner C . Conceptual framework of women's food environments and determinants of food acquisition and dietary intake in low- and middle-income countries: a scoping review. Lancet Planet Health. (2025) 9:101280. doi: 10.1016/j.lanplh.2025.06.004 40652946

[42] Neufeld LM Andrade EB Ballonoff Suleiman A Barker M Beal T Blum LS . Food choice in transition: adolescent autonomy, agency, and the food environment. Lancet. (2022) 399:185–97. doi: 10.1016/S0140-6736(21)01687-1 34856191

[43] Munawaroh RrS Maladi M Boedi S. Exploring the role of social capital in traditional market survival amid urban retail transformation. Asian J Econ Busin Acc. (2025) 25:53–8. doi: 10.9734/ajeba/2025/v25i51784

[44] Van Eck E. “That market has no quality”: performative place frames, racialisation, and affective re-inscriptions in an outdoor retail market in Amsterdam. Trans Inst British Geog. (2022) 47:547–61. doi: 10.1111/tran.12515

[45] Rodriguez-Lozada P Torres-Chaupiz M Grillo-Torres C Cordova-Buiza F. Consumer behaviour in supermarkets and food markets: a mixed study in Peru. IBIMABR. (2024) 2024. doi: 10.5171/2024.686604

[46] Ravikumar D Spyreli E Woodside J McKinley M Kelly C. Parental perceptions of the food environment and their influence on food decisions among low-income families: a rapid review of qualitative evidence. BMC Public Health. (2022) 22:9. doi: 10.1186/s12889-021-12414-z 34983469 PMC8727174

[47] Vos M Deforche B Van Kerckhove A Michels N Poelman M Geuens M . Determinants of healthy and sustainable food choices in parents with a higher and lower socioeconomic status: a qualitative study. Appetite. (2022) 178:106180. doi: 10.1016/j.appet.2022.106180 35863506

[48] Van Der Heijden A Wiggins S. Interaction as the foundation for eating practices in shared mealtimes. Appetite. (2025) 205:107585. doi: 10.1016/j.appet.2024.107585 38945367

[49] McCoy CA Johnston E Hogan C. The impact of socioeconomic status on health practices via health lifestyles: results of qualitative interviews with Americans from diverse socioeconomic backgrounds. Soc Sci Med. (2024) 344:116618. doi: 10.1016/j.socscimed.2024.116618 38324976

[50] Djojosoeparto SK Kamphuis CBM Vandevijvere S Murrin C Stanley I Romaniuk P . Strength of EU-level food environment policies and priority recommendations to create healthy food environments. Eur J Public Health. (2022) 32:504–11. doi: 10.1093/eurpub/ckac010 35265982 PMC9159309

