# Supplementary material for: The social production of the food environment: a mixed-methods analysis of the unequal distribution of capitals and competencies in a Latin American metropolis
Source: Front Nutr. 2026 Jul 10;13:1823248. doi: 10.3389/fnut.2026.1823248 (PMC13397664; doi:10.3389/fnut.2026.1823248)
Supplement: Supplementary file 1 [file Table_1.docx]

Supplementary Material

**Table 1. Question 11. When purchasing food, how important is it to you…? By district. Percentage (frequency).**

| Statement | High SES | | | Low SES | | | p-value |
| --- | --- | --- | --- | --- | --- | --- | --- |
|  | **Not important** | **Somewhat important** | **Very important** | **Not important** | **Somewhat important** | **Very important** |  |
| a. Flavor | 2.3% (9) | 12.6% (50) | 85.1% (338) | 1.3% (5) | 13.9% (54) | 84.8% (329) |  |
| b. Nutrition | 2.8% (11) | 21.4% (85) | 75.8% (301) | 4.9% (19) | 18.6% (72) | 76.5% (297) |  |
| c. Price | 7.6% (30) | 34.8% (138) | 57.7% (229) | 5.9% (23) | 14.9% (58) | 79.1% (307) | < 0.005* |
| d. Ease of preparation | 22.7% (90) | 37.8% (150) | 39.5% (157) | 14.4% (56) | 23.7% (92) | 61.9% (240) | < 0.005* |
| e. Weight control | 24.7% (98) | 35.3% (140) | 40.1% (159) | 10.3% (40) | 22.2% (86) | 67.5% (262) | < 0.005* |

*Chi-Square. †Chi-Square (Monte Carlo) analysis of adjusted standardized residuals (Haberman's function). The subscript **a** indicates that the observed frequency is significantly higher than the expected frequency. The subscript **b** indicates that the observed frequency is significantly lower than the expected frequency (*p* < .05).

Table 2. Question 12. Does the presence of warning labels influence your purchase?, by district. Percentage (Frequency)

| Response | High SES | Low SES | p-value |
| --- | --- | --- | --- |
| a. Yes, it influences; I choose foods with fewer warning labels | 32.7% (130) | 37.4% (145) | < 0.005* |
| b. Yes, it influences; I do not buy foods with warning labels | 5.8% (23)^b^ | 13.7% (53) |  |
| c. Yes, it influences; I buy less than I would have bought if the product had no warning labels | 29.5% (117)^a^ | 14.2% (55) |  |
| d. Not influence | 32.0% (127) | 34.8% (135) |  |

*Chi-Square. †Chi-Square (Monte Carlo) analysis of adjusted standardized residuals (Haberman's function). The subscript **a** indicates that the observed frequency is significantly higher than the expected frequency. The subscript **b** indicates that the observed frequency is significantly lower than the expected frequency (*p* < .05).

**Table 3. Question 13. Perception when seeing a food product with one or more warning labels, by district. Percentage (Frequency)**

| Response | High SES | Low SES | p-value |
| --- | --- | --- | --- |
| a. I should not buy it | 19.6% (78)^b^ | 32.2% (125) | < 0.005* |
| b. I should buy it less often | 29.0% (115)^a^ | 18.6% (72) |  |
| c. I should buy it in smaller quantities | 27.5% (109) | 22.2% (86) |  |
| d. I am indifferent | 23.9% (95) | 27.1% (105) |  |

*Chi-Square. †Chi-Square (Monte Carlo) analysis of adjusted standardized residuals (Haberman's function). The subscript **a** indicates that the observed frequency is significantly higher than the expected frequency. The subscript **b** indicates that the observed frequency is significantly lower than the expected frequency (*p* < .05).

**Table 4. Question 7a. Places where the majority of food is purchased, by district. Percentage (Frequency)**

| Location | High SES | | Low SES | | p-value |
| --- | --- | --- | --- | --- | --- |
|  | Yes | No | Yes | No |  |
| Supermarket | 93.7% (372)^a^ | 6.3% (25)^b^ | 85.3% (331) | 14.7% (57) | < 0.005* |
| Convenience store (OK Market, Gas station store) | 1.0% (4) | 99.0% (393) | 1.0% (4) | 99.0% (384) |  |
| Bakery | 14.6% (58)b | 85.4% (339)^a^ | 24.7% (96) | 75.3% (292) | < 0.005* |
| Corner store, grocery store, or greengroce | 37.0% (147)^b^ | 63.0% (250)^a^ | 45.1% (175) | 54.9% (213) | 0.021* |
| farmers' market | 55.2% (219)^b^ | 44.8% (178)^a^ | 85.3% (331) | 14.7% (57) | < 0.005* |
| Wholesale market | 3.0% (12)^b^ | 97.0% (385)^a^ | 16.2% (63) | 83.8% (325) | < 0.005* |
| Butcher shop | 7.1% (28)^b^ | 92.9% (369)^a^ | 34.0% (132) | 66.0% (256) | < 0.005* |
| Other (please specify) | 4.0% (16) | 96.0% (381) | 3.6% (14) | 96.4% (374) |  |

*Chi-Square. †Chi-Square (Monte Carlo) analysis of adjusted standardized residuals (Haberman's function). The subscript **a** indicates that the observed frequency is significantly higher than the expected frequency. The subscript **b** indicates that the observed frequency is significantly lower than the expected frequency (*p* < .05).

**Table 5. Question 7b. Most important place where the majority of food is purchased, by district. Percentage (Frequency)**

| **Location** | High SES | Low SES | p-value |
| --- | --- | --- | --- |
| Supermarket | 73.6% (292)^a^ | 51.0% (198) | < 0.005† |
| Convenience store (OK Market, Gas station store) | 0.0% (0) | 0.0% (0) |  |
| Bakery | 0.0% (0) | 0.3% (1) |  |
| Corner store, grocery store, or greengroce | 5.0% (20) | 7.0% (27) |  |
| farmers' market | 18.1% (72)^b^ | 31.4% (122) |  |
| Wholesale market | 1.5% (6)^b^ | 7.0% (27) |  |
| Butcher shop | 0.0% (0)^b^ | 1.0% (4) |  |
| Other (please specify) | 1.8% (7) | 2.3% (9) |  |

*Chi-Square. †Chi-Square (Monte Carlo) analysis of adjusted standardized residuals (Haberman's function). The subscript **a** indicates that the observed frequency is significantly higher than the expected frequency. The subscript **b** indicates that the observed frequency is significantly lower than the expected frequency (*p* < .05).

**Table 6. Question 8. Walking time to the place where the majority of food is purchased, by district. Percentage (Frequency)**

| Time | High SES | Low SES | p-value |
| --- | --- | --- | --- |
| 10 minutes or less | 60.5% (240)^a^ | 47.9% (186) | < 0.005* |
| 11 to 20 minutes | 19.4% (77) | 25.0% (97) |  |
| 21 to 30 minutes | 5.5% (22)^b^ | 10.3% (40) |  |
| More than 30 minutes | 14.6% (58) | 16.8% (65) |  |

*Chi-Square. †Chi-Square (Monte Carlo) analysis of adjusted standardized residuals (Haberman's function). The subscript **a** indicates that the observed frequency is significantly higher than the expected frequency. The subscript **b** indicates that the observed frequency is significantly lower than the expected frequency (*p* < .05).

**Table 7. Question 6. Perception of food within the neighborhood, by district. Percentage (Frequency)**

| Statement | High SES | | | | | Low SES | | | | | p-value |
| --- | --- | --- | --- | --- | --- | --- | --- | --- | --- | --- | --- |
|  | Never | Occasionally | Almost Always | Always | DK/NA | Never | Occasionally | Almost Always | Always | DK/NA |  |
| a. It is easy to buy fresh fruits and vegetables in my neighborhood. | 1.2% (5)^b^ | 7.3% (29)^b^ | 15.4% (61) | 76.1% (302)^a^ | 0.0% (0) | 6.2% (24) | 14.4% (56) | 19.6% (76) | 59.8% (232) | 0.0% (0) | < 0.005* |
| b. The fresh foods sold in my neighborhood are of good quality. | 0.8% (3)^b^ | 6.0% (24)^b^ | 22.9% (91) | 69.8% (277)^a^ | 0.5% (2) | 3.6% (14) | 12.6% (49) | 26.6% (103) | 56.2% (218) | 1.0% (4) | < 0.005† |
| c. A great variety of fresh fruits and vegetables can be found in my neighborhood. | 2.0% (8)^b^ | 10.8% (43) | 19.9% (79) | 67.0% (266)^a^ | 0.3% (1) | 8.5% (33) | 14.4% (56) | 18.8% (73) | 58.0% (225) | 0.3% (1) | < 0.005† |
| d. In my neighborhood, it is easy to buy packaged foods without any black warning labels (High in). | 10.8% (43)^b^ | 26.4% (105)^a^ | 27.5% (109)^a^ | 26.7% (106)^a^ | 8.6% (34) | 61.1% (237) | 15.5% (60) | 8.5% (33) | 8.0% (31) | 6.9% (27) | < 0.005* |
| e. The packaged foods in my neighborhood are of good quality. | 1.8% (7)^b^ | 7.0% (28)^b^ | 26.9% (107)^b^ | 60.5% (240)^a^ | 3.8% (15) | 9.3% (36) | 18.3% (71) | 36.9% (143) | 30.4% (118) | 5.1% (20) | < 0.005* |
| f. A great variety of packaged foods without any black warning labels (High in) can be found in my neighborhood. | 14.1% (56)^b^ | 28.0% (111)^a^ | 26.4% (105)^a^ | 23.7% (94)^a^ | 7.8% (31) | 61.9% (240) | 17.3% (67) | 6.4% (25) | 7.7% (30) | 6.7% (26) | < 0.005* |

*Chi-Square. †Chi-Square (Monte Carlo) analysis of adjusted standardized residuals (Haberman's function). The subscript **a** indicates that the observed frequency is significantly higher than the expected frequency. The subscript **b** indicates that the observed frequency is significantly lower than the expected frequency (*p* < .05).

Table 8. Question 9. Ease or difficulty of obtaining food at the place where the majority of food is purchased, by district. Percentage (Frequency)

| **Food Item** | High SES | | | | Low SES | | | | | p-value |  |
| --- | --- | --- | --- | --- | --- | --- | --- | --- | --- | --- | --- |
|  | **Very Difficult** | **Difficult** | **Easy** | **Very Easy** | **Very Difficult** | **Difficult** | **Easy** | **Very Easy** |  | | |
| a. Fresh or frozen fruits and vegetables | 0.8%  (3)^b^ | 3.0% (12)^b^ | 33.5% (133) | 62.7% (249)^a^ | 7.2% (28) | 9.3% (36) | 37.9% (147) | 45.6% (177) | < 0.005* | | |
| c. Canned, fresh, or frozen fish | 1.8%  (7)^b^ | 8.8% (35) | 41.8% (166) | 47.6% (189)^a^ | 7.0% (27) | 12.6% (49) | 43.8% (170) | 36.6% (142) | < 0.005* | | |
| d. Sweets and salty snacks | 3.0% (12) | 6.8% (27)^a^ | 21.2% (84)^b^ | 69.0% (274)^a^ | 2.8% (11) | 2.1%  (8) | 36.1% (140) | 59.0% (229) | < 0.005* | | |
| f. Sugar-sweetened sodas or other sugary drinks (sports drinks, juices, nectars, etc.) | 5.5% (22)^a^ | 5.0% (20) | 15.9% (63)^b^ | 73.6% (292)^a^ | 1.8%  (7) | 3.1% (12) | 29.1% (113) | 66.0% (256) | < 0.005* | | |

*Chi-Square. †Chi-Square (Monte Carlo) analysis of adjusted standardized residuals (Haberman's function). The subscript **a** indicates that the observed frequency is significantly higher than the expected frequency. The subscript **b** indicates that the observed frequency is significantly lower than the expected frequency (*p* < .05).

**Table 9. Question 1. Food items available at home during the past week, by district. Percentage (Frequency)**

| Food Item | High SES | | Low SES | | p-value |
| --- | --- | --- | --- | --- | --- |
|  | Yes | **No** | Yes | **No** |  |
| a. Fruits such as orange, banana, apple, pear, peach | 97.0% (385) | 3.0% (12) | 94.8% (368) | 5.2% (20) |  |
| b. Frozen ready meals such as pizza, lasagna, empanadas | 20.4% (81) | 79.6% (316) | 15.2% (59) | 84.8% (329) |  |
| cc. Vegetables such as spinach, chard, broccoli, cauliflower, zucchini, beetroot | 83.6% (332) | 16.4% (65) | 87.4% (339) | 12.6% (49) |  |
| d. Soft drinks (exclude unflavored mineral water) | 59.9% (238) | 40.1% (159) | 62.1% (241) | 37.9% (147) |  |
| e. Fruits such as mango, blueberries, blackberries, raspberries, persimmon, pomegranate | 44.8% (178) | 55.2% (219)^a^ | 28.4% (110) | 71.6% (278) | < 0.005* |
| f. Sweets (candies) and chocolates | 61.2% (243) | 38.8% (154)^a^ | 53.9% (209) | 46.1% (179) | 0.037* |
| g. Vegetables such as lettuce, cabbage, celery, tomato, onion, carrot | 98.5% (391) | 1.5% (6) | 98.7% (383) | 1.3% (5) |  |
| h. Ice cream (tubs or individual) | 42.3% (168) | 57.7% (229)^a^ | 33.0% (128) | 67.0% (260) | 0.007* |
| i. Dairy such as milk (unflavored), fresh cheese, "quesillo", yellow cheese, yogurt, or other unsweetened dairy. | 94.7% (376) | 5.3% (21) | 94.3% (366) | 5.7% (22) |  |
| j. Unsalted or unsweetened nuts such as almonds, peanuts, and walnuts | 78.6% (312) | 21.4% (85)^a^ | 62.4% (242) | 37.6% (146) | < 0.005* |
| k. Cold cuts (pork ham, salami, "jamonada", pâté, etc.) | 66.5% (264) | 33.5% (133)^b^ | 73.5% (285) | 26.5% (103) | 0.033* |
| l. Legumes (beans, lentils, chickpeas, etc.) dried or canned | 91.4% (363) | 8.6% (34) | 91.2% (354) | 8.8% (34) |  |
| m. Sweet or cream-filled cookies | 49.6% (197) | 50.4% (200) | 53.1% (206) | 46.9% (182) |  |
| n. Processed meats or sausages (hot dogs, "longanizas", "choricillos", burgers, etc.) | 57.7% (229) | 42.3% (168) | 53.4% (207) | 46.6% (181) |  |
| ñ. Vegetables such as arugula, watercress, Brussels sprouts, kale, endive, eggplant | 43.1% (171) | 56.9% (226)^a^ | 27.3% (106) | 72.7% (282) | < 0.005* |
| o. Pastries, cakes, or sweet doughs | 35.8% (142) | 64.2% (255) | 38.9% (151) | 61.1% (237) |  |
| p. Salty snacks (potato chips, corn sticks, or others) | 50.1% (199) | 49.9% (198) | 37.1% (144)^a^ | 62.9% (244) | < 0.005* |

*Chi-Square. †Chi-Square (Monte Carlo) analysis of adjusted standardized residuals (Haberman's function). The subscript **a** indicates that the observed frequency is significantly higher than the expected frequency. The subscript **b** indicates that the observed frequency is significantly lower than the expected frequency (*p* < .05).

**Table 10. Question 5. Frequency of food consumption, by district. Percentage (Frequency)**

| Food Item | High SES | | | | | Low SES | | | | | | p-value |
| --- | --- | --- | --- | --- | --- | --- | --- | --- | --- | --- | --- | --- |
|  | **< Once a week / Never** | **1-2 times a week** | **3-4 times a week** | **5-6 times a week** | **Every day** | | **< Once a week / Never** | **1-2 times a week** | **3-4 times a week** | **5-6 times a week** | **Every day** |  |
| a. Fruit, not counting natural juices | 4.0% (16) | 18.7% (74) | 24.4% (97) | 11.3% (45) | 41.6% (165) | | 9.0% (35) | 17.0% (66) | 21.9% (85) | 9.6% (37) | 42.5% (165) |  |
| b. Natural fruit juices, such as orange, grapefruit, or peach juice (without added sugar) | 57.9% (230) | 20.9% (83) | 10.1% (40) | 2.0% (8) | 9.1% (36) | | 64.2% (249) | 16.7% (65) | 8.0% (31) | 3.9% (15) | 7.2% (28) |  |
| c. Salads (excluding those containing mainly potatoes, rice, or noodles) | 2.0% (8) | 10.8% (43) | 19.4% (77) | 13.1% (52) | 54.7% (217) | | 153.9% (15) | 11.1% (43) | 14.7% (57) | 10.8% (42) | 59.5% (231) |  |
| d. Vegetables, in stews or cooked | 8.8% (35)^b^ | 31.2% (124)^b^ | 28.5% (113)^a^ | 14.4% (57)^a^ | 17.1% (68) | | 15.7% (61) | 39.4% (153) | 17.3% (67) | 9.3% (36) | 18.3% (71) | < 0.005* |

*Chi-Square. †Chi-Square (Monte Carlo) analysis of adjusted standardized residuals (Haberman's function). The subscript **a** indicates that the observed frequency is significantly higher than the expected frequency. The subscript **b** indicates that the observed frequency is significantly lower than the expected frequency (*p* < .05).

**Table 11. Question 2. Household perception of aspects related to food, by district. Percentage (Frequency)**

| Aspect | High SES | | | | | Low SES | | | | | p-value |
| --- | --- | --- | --- | --- | --- | --- | --- | --- | --- | --- | --- |
|  | Very Bad | Bad | Fair | Good | Very Good | Very Bad | Bad | Fair | Good | Very Good |  |
| a. Physical location where food is consumed (e.g., dining room, kitchen, bedroom) | 0.2% (1) | 0.8% (3) | 7.1% (28) | 42.8% (170)^b^ | 49.1% (195)^a^ | 0.5% (2) | 1.5% (6) | 7.2% (28) | 63.7% (247 | 27.1% (105) | < 0.005* |
| b. Time allocated to food (preparation and consumption) | 1.3% (5) | 4.5% (18) | 18.1% (72) | 48.6% (193)^b^ | 27.5% (109)^a^ | 0.5% (2) | 3.1% (12) | 22.7% (88) | 58.5% (227) | 15.2% (59) | < 0.005* |
| c. Food consumption schedules | 2.5% (10) | 3.5% (14) | 25.4% (101) | 47.4% (188)^b^ | 21.2% (84)^a^ | 1.6% (6) | 5.9% (23) | 24.2% (94) | 57.0% (221) | 11.3% (44) | < 0.005* |

*Chi-Square. †Chi-Square (Monte Carlo) analysis of adjusted standardized residuals (Haberman's function). The subscript **a** indicates that the observed frequency is significantly higher than the expected frequency. The subscript **b** indicates that the observed frequency is significantly lower than the expected frequency (*p* < .05).

**Table 12. Question 3. Frequency with which household members eat together, by district. Percentage (Frequency)**

| Meal | High SES | | | | | | | Low SES | | | | | | p-value |
| --- | --- | --- | --- | --- | --- | --- | --- | --- | --- | --- | --- | --- | --- | --- |
|  | **Never** | **Occasionally** | **Almost Always** | **Always** | **Does not have this meal** | **Lives alone** | **Never** | | **Occasionally** | **Almost Always** | **Always** | **Does not have this meal** | **Lives alone** |  |
| a. Breakfast | 19.1% (76) | 32.2% (128) | 12.6% (50) | 15.6% (62)^b^ | 0.8% (3) | 19.7% (78)^a^ | 17.8% (69) | | 36.9% (143) | 13.4% (52) | 23.4% (91) | 0.5% (2) | 8.0% (31) | < 0.005^†^ |
| b. Lunch | 10.3% (41) | 35.3% (140) | 16.9% (67) | 17.6% (70)^b^ | 0.3% (1) | 19.6% (78)^a^ | 9.8% (38) | | 37.4% (145) | 14.4% (56) | 30.4% (118) | 0.0% (0) | 8.0% (31) | < 0.005* |
| c. "Once" (Afternoon snack/tea) | 8.3% (33) | 16.6% (66) | 16.9% (67) | 23.4% (93)^b^ | 15.6% (62)^a^ | 19.2% (76)^a^ | 8.2% (32) | | 16.0% (62) | 15.0% (58) | 46.1% (179) | 7.0% (27) | 7.7% (30) | < 0.005† |
| d. Dinner | 3.1% (12)^b^ | 7.2% (28) | 11.9% (46)^a^ | 23.5% (91) | 36.7% (142)^b^ | 17.6% (68)^a^ | 10.0% (39) | | 4.9% (19) | 3.9% (15) | 18.8% (73) | 57.0% (221) | 5.4% (21 | < 0.005* |

*Chi-Square. †Chi-Square (Monte Carlo) analysis of adjusted standardized residuals (Haberman's function). The subscript **a** indicates that the observed frequency is significantly higher than the expected frequency. The subscript **b** indicates that the observed frequency is significantly lower than the expected frequency (*p* < .05).

**Table 13. Question 4. Frequency with which household members eat in front of the television, by district. Percentage (Frequency)**

| Meal | High SES | | | | | Low SES | | | | | p-value |
| --- | --- | --- | --- | --- | --- | --- | --- | --- | --- | --- | --- |
|  | **Never** | **Occasionally** | **Almost Always** | **Always** | **Does not have this meal** | **Never** | **Occasionally** | **Almost Always** | **Always** | **Does not have this meal** |  |
| a. Breakfast | 45.6% (181)^a^ | 19.4% (77) | 15.4% (61) | 18.6% (74)^b^ | 1.0% (4) | 37.9% (147) | 17.3% (67) | 13.1% (51) | 31.4% (122) | 0.3% (1) | < 0.005† |
| b. Lunch | 41.3% (164)^a^ | 25.9% (103)^a^ | 12.8% (51) | 19.7% (78)^b^ | 0.3% (1) | 32.5% (126) | 18.5% (72) | 15.5% (60) | 33.2% (129) | 0.3% (1) | < 0.005* |
| c. "Once" (Afternoon snack/tea) | 26.7% (106) | 21.1% (84)^a^ | 15.9% (63) | 18.4% (73)^b^ | 17.9% (71)^a^ | 27.8% (108) | 14.2% (55) | 13.4% (52) | 37.6% (146) | 7.0% (27) | < 0.005† |
| d. Dinner | 20.4% (81) | 13.1% (52)^a^ | 12.1% (48)^a^ | 13.6% (54) | 40.8% (162)^b^ | 20.9% (81) | 5.7% (22) | 4.9% (19) | 13.4% (52) | 55.1% (214) | < 0.005* |

*Chi-Square. †Chi-Square (Monte Carlo) analysis of adjusted standardized residuals (Haberman's function). The subscript **a** indicates that the observed frequency is significantly higher than the expected frequency. The subscript **b** indicates that the observed frequency is significantly lower than the expected frequency (*p* < .05).
