# Supplementary material for: The social production of the food environment: a mixed-methods analysis of the unequal distribution of capitals and competencies in a Latin American metropolis
Source: Front Nutr. 2026 Jul 10;13:1823248. doi: 10.3389/fnut.2026.1823248 (PMC13397664; doi:10.3389/fnut.2026.1823248)
Supplement: Supplementary file 2 [file Table_2.docx]

Supplementary Material

**
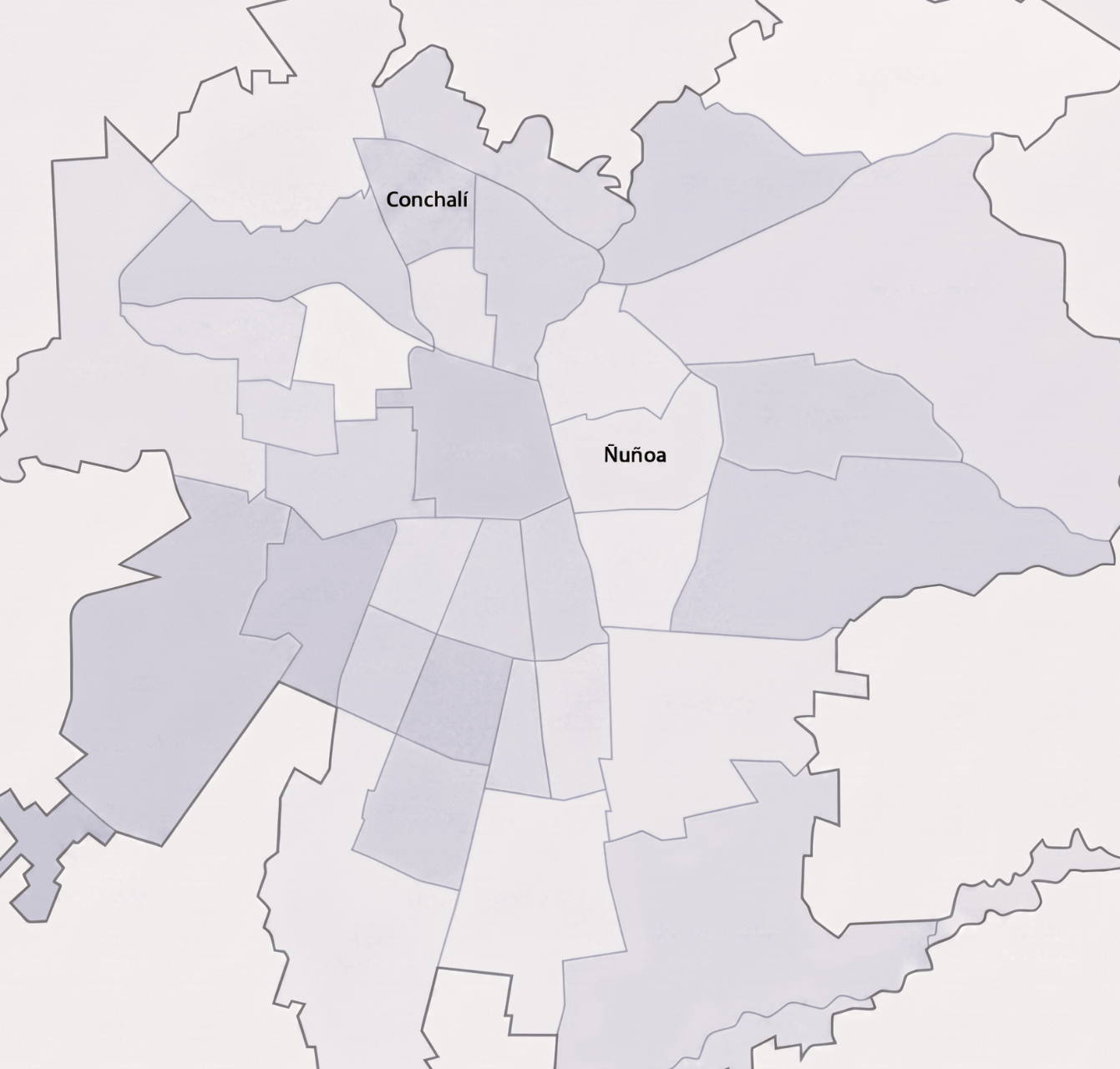
Figure S1. Location of study districts in the metropolitan area of Santiago, Chile: Ñuñoa (high socioeconomic status) and Conchalí (low socioeconomic status), based on multidimensional poverty criteria**

**Table S1.** Interpretive framework linking theoretical constructs to empirical evidence.

| Construct | Interpreted as | Empirical evidence |
| --- | --- | --- |
| Economic capital (Bourdieu) | Material constraints, price sensitivity, capacity for choice | NEMS-P Q11c; FG: “I stretch them like gum,” “what I have in money, I have to invest in the children” |
| Social capital (Bourdieu) | Trust networks, informal credit, vendor relationships | NEMS-P Q7a; FG: “casero,” “fiado,” “we pay on the fortnight” |
| Habitus (Bourdieu) | Embodied dispositions toward norms and tastes | NEMS-P Q1201313; FG: “it is my health, but026,” “it is an exquisite celery” |
| Materialities (Shove) | Infrastructure, available money, products, physical space | NEMS-P Q7a, Q8; FG: “five lucas,” “I have the supermarket mapped” |
| Competencies (Shove) | Skills for planning, negotiation, evaluation | FG: “I already know which are the aisles,” “I stretch them like gum” |
| Meanings (Shove) | Values, aspirations, senses attributed to food | FG: “it019s spectacular,” “it019s like a social life,” “necessity, saving, and subsistence,” “shopping is a chore” |

*Note.* NEMS-P = Nutrition Environment Measure Scale013Perceptions adapted for Chile. Q11c = importance of price when purchasing; Q7a = places where food is purchased; Q8 = walking time to main purchase place; Q1201313 = attitudes toward front-of-pack warning labels. When a construct has no direct NEMS-P indicator, only focus group evidence is listed.

**Table S2. Question 11. When purchasing food, how important is it to you…? By district. Percentage (frequency).**

| Statement | High SES | | | Low SES | | | p-value |
| --- | --- | --- | --- | --- | --- | --- | --- |
|  | **Not important** | **Somewhat important** | **Very important** | **Not important** | **Somewhat important** | **Very important** |  |
| a. Flavor | 2.3% (9) | 12.6% (50) | 85.1% (338) | 1.3% (5) | 13.9% (54) | 84.8% (329) |  |
| b. Nutrition | 2.8% (11) | 21.4% (85) | 75.8% (301) | 4.9% (19) | 18.6% (72) | 76.5% (297) |  |
| c. Price | 7.6% (30) | 34.8% (138) | 57.7% (229) | 5.9% (23) | 14.9% (58) | 79.1% (307) | < 0.005* |
| d. Ease of preparation | 22.7% (90) | 37.8% (150) | 39.5% (157) | 14.4% (56) | 23.7% (92) | 61.9% (240) | < 0.005* |
| e. Weight control | 24.7% (98) | 35.3% (140) | 40.1% (159) | 10.3% (40) | 22.2% (86) | 67.5% (262) | < 0.005* |

*Chi-Square. The subscript **a** indicates that the observed frequency is significantly higher than the expected frequency. The subscript **b** indicates that the observed frequency is significantly lower than the expected frequency (*p* < .05).

**Table S3. Question 13. Perception when seeing a food product with one or more warning labels, by district. Percentage (Frequency)**

| Response | High SES | Low SES | p-value |
| --- | --- | --- | --- |
| a. I should not buy it | 19.6% (78)^b^ | 32.2% (125) | < 0.005* |
| b. I should buy it less often | 29.0% (115)^a^ | 18.6% (72) |  |
| c. I should buy it in smaller quantities | 27.5% (109) | 22.2% (86) |  |
| d. I am indifferent | 23.9% (95) | 27.1% (105) |  |

*Chi-Square. The subscript **a** indicates that the observed frequency is significantly higher than the expected frequency. The subscript **b** indicates that the observed frequency is significantly lower than the expected frequency (*p* < .05).

**Table S4. Question 7b. Most important place where the majority of food is purchased, by district. Percentage (Frequency)**

| **Location** | High SES | Low SES | p-value |
| --- | --- | --- | --- |
| Supermarket | 73.6% (292)^a^ | 51.0% (198) | < 0.005† |
| Convenience store (OK Market, Gas station store) | 0.0% (0) | 0.0% (0) |  |
| Bakery | 0.0% (0) | 0.3% (1) |  |
| Corner store, grocery store, or greengroce | 5.0% (20) | 7.0% (27) |  |
| farmers' market | 18.1% (72)^b^ | 31.4% (122) |  |
| Wholesale market | 1.5% (6)^b^ | 7.0% (27) |  |
| Butcher shop | 0.0% (0)^b^ | 1.0% (4) |  |
| Other (please specify) | 1.8% (7) | 2.3% (9) |  |

*Chi-Square. †Chi-Square (Monte Carlo) analysis of adjusted standardized residuals (Haberman's function). The subscript **a** indicates that the observed frequency is significantly higher than the expected frequency. The subscript **b** indicates that the observed frequency is significantly lower than the expected frequency (*p* < .05).

**Table S5. Question 8. Walking time to the place where the majority of food is purchased, by district. Percentage (Frequency)**

| Time | High SES | Low SES | p-value |
| --- | --- | --- | --- |
| 10 minutes or less | 60.5% (240)^a^ | 47.9% (186) | < 0.005* |
| 11 to 20 minutes | 19.4% (77) | 25.0% (97) |  |
| 21 to 30 minutes | 5.5% (22)^b^ | 10.3% (40) |  |
| More than 30 minutes | 14.6% (58) | 16.8% (65) |  |

*Chi-Square. The subscript **a** indicates that the observed frequency is significantly higher than the expected frequency. The subscript **b** indicates that the observed frequency is significantly lower than the expected frequency (*p* < .05).

**Table S6. Question 9. Ease or difficulty of obtaining food at the place where the majority of food is purchased, by district. Percentage (Frequency)**

| **Food Item** | High SES | | | | Low SES | | | | | p-value |  |
| --- | --- | --- | --- | --- | --- | --- | --- | --- | --- | --- | --- |
|  | **Very Difficult** | **Difficult** | **Easy** | **Very Easy** | **Very Difficult** | **Difficult** | **Easy** | **Very Easy** |  | | |
| a. Fresh or frozen fruits and vegetables | 0.8%  (3)^b^ | 3.0% (12)^b^ | 33.5% (133) | 62.7% (249)^a^ | 7.2% (28) | 9.3% (36) | 37.9% (147) | 45.6% (177) | < 0.005* | | |
| c. Canned, fresh, or frozen fish | 1.8%  (7)^b^ | 8.8% (35) | 41.8% (166) | 47.6% (189)^a^ | 7.0% (27) | 12.6% (49) | 43.8% (170) | 36.6% (142) | < 0.005* | | |
| d. Sweets and salty snacks | 3.0% (12) | 6.8% (27)^a^ | 21.2% (84)^b^ | 69.0% (274)^a^ | 2.8% (11) | 2.1%  (8) | 36.1% (140) | 59.0% (229) | < 0.005* | | |
| f. Sugar-sweetened sodas or other sugary drinks (sports drinks, juices, nectars, etc.) | 5.5% (22)^a^ | 5.0% (20) | 15.9% (63)^b^ | 73.6% (292)^a^ | 1.8%  (7) | 3.1% (12) | 29.1% (113) | 66.0% (256) | < 0.005* | | |

*Chi-Square. The subscript **a** indicates that the observed frequency is significantly higher than the expected frequency. The subscript **b** indicates that the observed frequency is significantly lower than the expected frequency (*p* < .05).

**Table S7. Question 5. Frequency of food consumption, by district. Percentage (Frequency)**

| Food Item | High SES | | | | | Low SES | | | | | | p-value |
| --- | --- | --- | --- | --- | --- | --- | --- | --- | --- | --- | --- | --- |
|  | **< Once a week / Never** | **1-2 times a week** | **3-4 times a week** | **5-6 times a week** | **Every day** | | **< Once a week / Never** | **1-2 times a week** | **3-4 times a week** | **5-6 times a week** | **Every day** |  |
| a. Fruit, not counting natural juices | 4.0% (16) | 18.7% (74) | 24.4% (97) | 11.3% (45) | 41.6% (165) | | 9.0% (35) | 17.0% (66) | 21.9% (85) | 9.6% (37) | 42.5% (165) |  |
| b. Natural fruit juices, such as orange, grapefruit, or peach juice (without added sugar) | 57.9% (230) | 20.9% (83) | 10.1% (40) | 2.0% (8) | 9.1% (36) | | 64.2% (249) | 16.7% (65) | 8.0% (31) | 3.9% (15) | 7.2% (28) |  |
| c. Salads (excluding those containing mainly potatoes, rice, or noodles) | 2.0% (8) | 10.8% (43) | 19.4% (77) | 13.1% (52) | 54.7% (217) | | 153.9% (15) | 11.1% (43) | 14.7% (57) | 10.8% (42) | 59.5% (231) |  |
| d. Vegetables, in stews or cooked | 8.8% (35)^b^ | 31.2% (124)^b^ | 28.5% (113)^a^ | 14.4% (57)^a^ | 17.1% (68) | | 15.7% (61) | 39.4% (153) | 17.3% (67) | 9.3% (36) | 18.3% (71) | < 0.005* |

*Chi-Square. The subscript **a** indicates that the observed frequency is significantly higher than the expected frequency. The subscript **b** indicates that the observed frequency is significantly lower than the expected frequency (*p* < .05).

**Table S8. Question 2. Household perception of aspects related to food, by district. Percentage (Frequency)**

| Aspect | High SES | | | | | Low SES | | | | | p-value |
| --- | --- | --- | --- | --- | --- | --- | --- | --- | --- | --- | --- |
|  | Very Bad | Bad | Fair | Good | Very Good | Very Bad | Bad | Fair | Good | Very Good |  |
| a. Physical location where food is consumed (e.g., dining room, kitchen, bedroom) | 0.2% (1) | 0.8% (3) | 7.1% (28) | 42.8% (170)^b^ | 49.1% (195)^a^ | 0.5% (2) | 1.5% (6) | 7.2% (28) | 63.7% (247 | 27.1% (105) | < 0.005* |
| b. Time allocated to food (preparation and consumption) | 1.3% (5) | 4.5% (18) | 18.1% (72) | 48.6% (193)^b^ | 27.5% (109)^a^ | 0.5% (2) | 3.1% (12) | 22.7% (88) | 58.5% (227) | 15.2% (59) | < 0.005* |
| c. Food consumption schedules | 2.5% (10) | 3.5% (14) | 25.4% (101) | 47.4% (188)^b^ | 21.2% (84)^a^ | 1.6% (6) | 5.9% (23) | 24.2% (94) | 57.0% (221) | 11.3% (44) | < 0.005* |

*Chi-Square. The subscript **a** indicates that the observed frequency is significantly higher than the expected frequency. The subscript **b** indicates that the observed frequency is significantly lower than the expected frequency (*p* < .05).

**Table S9. Question 3. Frequency with which household members eat together, by district. Percentage (Frequency)**

| Meal | High SES | | | | | | | Low SES | | | | | | p-value |
| --- | --- | --- | --- | --- | --- | --- | --- | --- | --- | --- | --- | --- | --- | --- |
|  | **Never** | **Occasionally** | **Almost Always** | **Always** | **Does not have this meal** | **Lives alone** | **Never** | | **Occasionally** | **Almost Always** | **Always** | **Does not have this meal** | **Lives alone** |  |
| a. Breakfast | 19.1% (76) | 32.2% (128) | 12.6% (50) | 15.6% (62)^b^ | 0.8% (3) | 19.7% (78)^a^ | 17.8% (69) | | 36.9% (143) | 13.4% (52) | 23.4% (91) | 0.5% (2) | 8.0% (31) | < 0.005^†^ |
| b. Lunch | 10.3% (41) | 35.3% (140) | 16.9% (67) | 17.6% (70)^b^ | 0.3% (1) | 19.6% (78)^a^ | 9.8% (38) | | 37.4% (145) | 14.4% (56) | 30.4% (118) | 0.0% (0) | 8.0% (31) | < 0.005* |
| c. "Once" (Afternoon snack/tea) | 8.3% (33) | 16.6% (66) | 16.9% (67) | 23.4% (93)^b^ | 15.6% (62)^a^ | 19.2% (76)^a^ | 8.2% (32) | | 16.0% (62) | 15.0% (58) | 46.1% (179) | 7.0% (27) | 7.7% (30) | < 0.005† |
| d. Dinner | 3.1% (12)^b^ | 7.2% (28) | 11.9% (46)^a^ | 23.5% (91) | 36.7% (142)^b^ | 17.6% (68)^a^ | 10.0% (39) | | 4.9% (19) | 3.9% (15) | 18.8% (73) | 57.0% (221) | 5.4% (21 | < 0.005* |

*Chi-Square. †Chi-Square (Monte Carlo) analysis of adjusted standardized residuals (Haberman's function). The subscript **a** indicates that the observed frequency is significantly higher than the expected frequency. The subscript **b** indicates that the observed frequency is significantly lower than the expected frequency (*p* < .05).

**Table S10. Question 4. Frequency with which household members eat in front of the television, by district. Percentage (Frequency)**

| Meal | High SES | | | | | Low SES | | | | | p-value |
| --- | --- | --- | --- | --- | --- | --- | --- | --- | --- | --- | --- |
|  | **Never** | **Occasionally** | **Almost Always** | **Always** | **Does not have this meal** | **Never** | **Occasionally** | **Almost Always** | **Always** | **Does not have this meal** |  |
| a. Breakfast | 45.6% (181)^a^ | 19.4% (77) | 15.4% (61) | 18.6% (74)^b^ | 1.0% (4) | 37.9% (147) | 17.3% (67) | 13.1% (51) | 31.4% (122) | 0.3% (1) | < 0.005† |
| b. Lunch | 41.3% (164)^a^ | 25.9% (103)^a^ | 12.8% (51) | 19.7% (78)^b^ | 0.3% (1) | 32.5% (126) | 18.5% (72) | 15.5% (60) | 33.2% (129) | 0.3% (1) | < 0.005* |
| c. "Once" (Afternoon snack/tea) | 26.7% (106) | 21.1% (84)^a^ | 15.9% (63) | 18.4% (73)^b^ | 17.9% (71)^a^ | 27.8% (108) | 14.2% (55) | 13.4% (52) | 37.6% (146) | 7.0% (27) | < 0.005† |
| d. Dinner | 20.4% (81) | 13.1% (52)^a^ | 12.1% (48)^a^ | 13.6% (54) | 40.8% (162)^b^ | 20.9% (81) | 5.7% (22) | 4.9% (19) | 13.4% (52) | 55.1% (214) | < 0.005* |

*Chi-Square. †Chi-Square (Monte Carlo) analysis of adjusted standardized residuals (Haberman's function). The subscript **a** indicates that the observed frequency is significantly higher than the expected frequency. The subscript **b** indicates that the observed frequency is significantly lower than the expected frequency (*p* < .05).
